# Effects of Deep Reductions in Energy Storage Costs on Highly Reliable Wind and Solar Electricity Systems

**DOI:** 10.1016/j.isci.2020.101484

**Published:** 2020-08-20

**Authors:** Fan Tong, Mengyao Yuan, Nathan S. Lewis, Steven J. Davis, Ken Caldeira

**Affiliations:** 1 Department of Global Ecology, Carnegie Institution for Science, Stanford, CA, USA; 2 Energy Analysis & Environmental Impacts Division, Lawrence Berkeley National Laboratory, Berkeley, CA, USA; 3 Division of Chemistry and Chemical Engineering, California Institute of Technology, Pasadena, CA, USA; 4 Department of Earth System Science, University of California, Irvine, Irvine, CA, USA

**Keywords:** Energy Resources, Energy Policy, Energy Engineering

## Abstract

We use 36 years (1980–2015) of hourly weather data over the contiguous United States (CONUS) to assess the impact of low-cost energy storage on highly reliable electricity systems that use only variable renewable energy (VRE; wind and solar photovoltaics). Even assuming perfect transmission of wind and solar generation aggregated over CONUS, energy storage costs would need to decrease several hundred-fold from current costs (to ∼$1/kWh) in fully VRE electricity systems to yield highly reliable electricity without extensive curtailment of VRE generation. The role of energy storage changes from high-cost storage competing with curtailment to fill short-term gaps between VRE generation and hourly demand to near-free storage serving as seasonal storage for VRE resources. Energy storage faces “double penalties” in VRE/storage systems: with increasing capacity, (1) the additional storage is used less frequently and (2) hourly electricity costs would become less volatile, thus reducing price arbitrage opportunities for the additional storage.

## Highlights

• As storage capacity increases, hourly electricity costs become less volatile.
• High-cost storage fills short-term gaps between renewable electricity generation and demand
• Near-free storage provides reliable seasonal storage for variable renewable electricity
• With increasing storage capacity, additional storage is used less frequently

## Graphical Abstract

**Figure:**
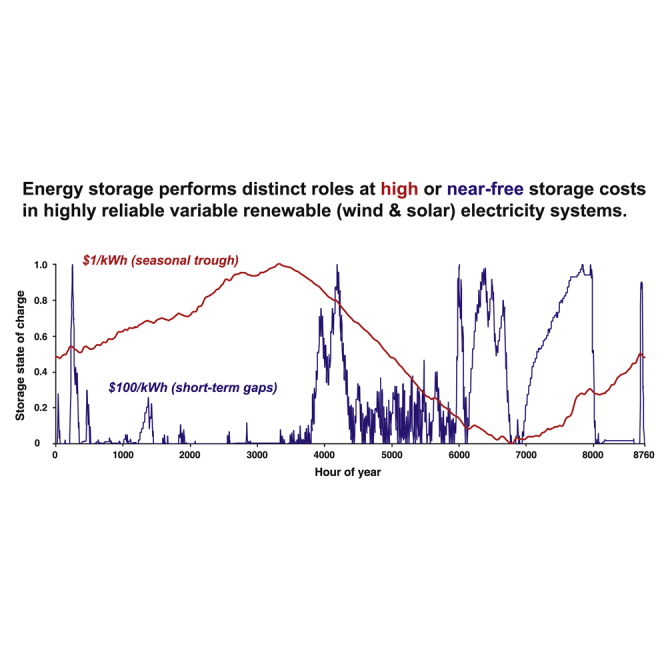

## Introduction

With a goal to reduce economic damages from future climate change (Davis et al., 2018;Electric Power Research Institute EPRI, 2018; Hoffert et al., 2002; Jacobson et al., 2015; Mai et al., 2018; Matthews and Caldeira, 2008; Wei et al., 2013), energy experts, regulators, policymakers, and the public are increasingly interested in, and often advocate for, electricity systems that rely primarily, if not exclusively, on variable renewable electricity (VRE; wind and solar photovoltaics) generation (Clack et al., 2017; Davis et al., 2018; de Sisternes et al., 2016; Denholm and Hand, 2011; Elliott, 2016; Gielen et al., 2019; Gilbraith et al., 2013; International Renewable Energy Agency (IRENA), 2017; Jacobson et al., 2015; Jenkins et al., 2018; Luderer et al., 2017, 2014; MacDonald et al., 2016; Mai et al., 2014; Mileva et al., 2016; Safaei and Keith, 2015; Sepulveda et al., 2018; Shaner et al., 2018; World Bank, 2019). Although many have argued that a broader portfolio of electricity generation technologies would more easily satisfy cost and performance requirements (Clack et al., 2017; Davis et al., 2018; Gilbraith et al., 2013; Hoffert et al., 2002; MacDonald et al., 2016; Sepulveda et al., 2018), considerable interest remains in systems that rely primarily on VRE technologies for electricity generation (de Sisternes et al., 2016; Denholm and Hand, 2011; Frew et al., 2016; Jacobson et al., 2015; Safaei and Keith, 2015; Shaner et al., 2018; Ziegler et al., 2019). To deal with the resource adequacy and reliability challenges for integrating VRE in electricity systems, a range of flexibility mechanisms have been proposed and studied. These mechanisms include balancing VRE generation over large geographic regions via transmission lines, curtailment of renewable electricity generation, grid-scale energy storage, cycling of dispatchable fossil fuel power plants, demand response, power-to-gas, power-to-liquids, and vehicle-grid integration (Blanco and Faaij, 2018; Braff et al., 2016; Brouwer et al., 2014; Castillo and Gayme, 2014; de Sisternes et al., 2016; Dowds et al., 2015; Frew et al., 2016; Kondziella and Bruckner, 2016; Lund et al., 2015; Milligan et al., 2017; Safaei and Keith, 2015; Zerrahn and Schill, 2017).

Cost-effective grid-scale energy storage is often considered as a critical enabling technology to realize an affordable, reliable electricity system based solely on VRE generation. Capital costs of energy storage technologies have decreased rapidly in conjunction with improvements in technical performance (Chen et al., 2009; Kittner et al., 2017; Schmidt et al., 2017) and development of new technologies (U.S. Department of Energy (DOE), 2018). Furthermore, energy storage technologies can play several roles in grid operations, such as energy balancing, price arbitrage, firming of capacity, and provision of ancillary services, thus increasing the value proposition of energy storage for electric power grids (Castillo and Gayme, 2014; Eyer and Corey, 2010; Schmidt et al., 2019; U.S. Department of Energy (DOE), 2011; U.S. Energy Information Administration (EIA), 2018a; Zakeri and Syri, 2015).

The technical performance and economic feasibility of energy storage in electricity systems have been evaluated using two distinct approaches. The first approach calculates the levelized cost of electricity (LCOE) in comparison with the economics of electricity generation technologies (Davis et al., 2018; Schmidt et al., 2019; Zakeri and Syri, 2015). Studies using this approach have often concluded that if energy storage costs were ∼$100/kWh (i.e., a factor of 2–5 reduction from current cost estimates that range from $200–500/kWh [Davis et al., 2018; Schmidt et al., 2019]), VRE generation supported by energy storage would be the cheapest way to produce electricity (Braff et al., 2016; Kittner et al., 2017). The second approach involves using complex power system models or energy systems models to simultaneously simulate or optimize the operation of generation technologies (such as wind and solar technologies) and energy storage technologies (such as batteries) (Clack et al., 2017; Connolly et al., 2010; de Sisternes et al., 2016; Denholm and Hand, 2011; Jacobson et al., 2015; Mileva et al., 2016; Safaei and Keith, 2015).

Variable renewable electricity with ∼$100/kWh energy storage could already deliver electricity at a lower LCOE than fossil-fuel-powered generators (Davis et al., 2018; Schmidt et al., 2019), but the resource adequacy and reliability of the electricity output from these systems may not be the same as those of existing electricity systems (Shaner et al., 2018). The North American Electricity Reliability Corporation (NERC) resource adequacy planning standard (ReliabilityFirst Corporation (RFC), 2017) requires a loss of load expectancy (LOLE) of <0.1 for the “integrated peak hour for all days of each planning year,” which has been clarified to mean that resource availability must be adequate to ensure that hourly averaged load is not met for no more than 1 h in 10 years. LCOE studies, although transparent and straightforward, are not designed to model resource adequacy or reliability of electricity systems (Khatib, 2016; U.S. Energy Information Administration (EIA), 2013). LCOE analyses also assume a predetermined operation model to amortize the capital investment over the useful life of the asset (e.g., energy storage).

Power system modeling studies provide increased capacity to model systems with high shares of variable renewable electricity (Connolly et al., 2010; Foley et al., 2010; Frew et al., 2019). MacDonald et al. (2016) showed that up to 80% reduction in carbon dioxide emissions could be achieved in an idealized electricity system that comprises substantial deployment of VRE capacity backed up by generators powered by natural gas, in conjunction with an expanded transmission network over the contiguous United States (CONUS). Detailed power system models and energy system models either explicitly model several flexibility mechanisms (which may or may not include energy storage) to ensure resource adequacy and reliability, or they simply assume resource adequacy is not an issue (Clack et al., 2017; Gilbraith et al., 2013; Jacobson et al., 2015; Jenkins et al., 2018; Sepulveda et al., 2018). As a result, grid-scale energy storage generally does not provide a significant role in such complex electricity grid systems. It is worth noting, however, that a study of the 2050 electricity grid in the United States found that energy storage would be necessary during sunset hours with high levels (>50%) of solar energy (Frew et al., 2019).

More recently, several studies investigated the role of energy storage to ensure highly reliable VRE electricity. Based on geophysical constraints, Shaner et al., 2018 showed that, without overbuilding VRE generation beyond the level to meet the mean electricity demand, a substantial capacity of energy storage (e.g., enough to meet electricity demand for several consecutive days or even weeks) would be needed to provide highly reliable electricity from VRE resources over a multi-decadal timescale. Using historical demand and wind generation with a specified capacity factor in Texas, U.S., Safaei and Keith (2015) concluded that seasonal energy storage was not economical at $100–$200/kWh energy storage costs. Based on an analysis of nearly 1,000 cases that covered varying CO_2_ limits and a range of future projected generation asset costs in two regional electricity systems (New England and Texas, U.S.), Sepulveda et al. (2018) concluded that availability of firm (i.e., dispatchable) low-carbon technologies would lower electricity costs relative to cases with solely VRE-based generation. Furthermore, Sepulveda et al. (2018) stated that, without firm low-carbon resources, a factor-of-two reduction in storage cost is not sufficient to affordably decarbonize the US power sector. Neither of these studies (Safaei and Keith, 2015; Sepulveda et al., 2018) assessed explicitly the costs at which energy storage would compete favorably with other generation technologies. While our work was underway, Ziegler et al. (2019) reported a study of 20 years of hourly resource variability at four locations in the U.S. and concluded that energy storage costs would need to be <$20/kWh to provide cost-competitive baseload electricity in resource-abundant locations such as Texas and Arizona, U.S. This study substantially refined the energy storage cost targets within the limitations of simplified yet artificial demand profiles and two selected state-wide regions of VRE generation. These prior studies (Safaei and Keith, 2015; Sepulveda et al., 2018; Ziegler et al., 2019) have not considered resource variability or geographical averaging of wind and solar resources over the CONUS or investigated the impact of deep reductions in energy storage cost on VRE/storage systems.

Several states and regions of the world have enacted legislation or set policy goals that specify that all electricity generation must come from zero-carbon or renewable resources before 2050 (State of California, 2018). The variability in wind and solar resources relative to demand must thus be addressed by storage, curtailment, or infrequent utilization of other zero-carbon generation technologies. Expensive grid-scale energy storage is often considered to be a primary technological barrier that precludes an affordable, reliable electricity system based solely on VRE generation in conjunction with storage (de Sisternes et al., 2016; Denholm and Hand, 2011; Elliott, 2016; International Renewable Energy Agency (IRENA), 2017; Mileva et al., 2016; Safaei and Keith, 2015; World Bank, 2019). However, the economics of energy storage depends critically on the frequency, magnitude, and duration of storage asset utilization, where storage competes with building additional VRE capacity to fill short-term and long-term gaps between VRE generation and electricity demand.

The focus of this study is to assess the effects of deep reductions in energy storage costs on highly reliable electricity systems based exclusively on VRE resources, using a transparent approach and decades-long hourly-resolution weather data. The fundamental trade-off in system design is between overbuilding VRE capacity (and thus curtailment of VRE electricity) and building and utilization of storage to mitigate the short-term and long-term variability between VRE resources and electricity demand. We used a standard yet simplified optimization model with parametrizations of a wide range of energy storage costs and considerations of VRE/storage system scenarios (in terms of technology availability and technology costs) to identify the roles of energy storage and the resulting system-level effects at different storage costs for the least-cost VRE/storage systems. Owing to abstraction, the absolute numbers in the results may be inaccurate when taken out of context, but relationships between VRE technologies and energy storage (e.g., ratios in terms of costs, capacity, and generation) in the least-cost systems could have practical implications for actual electricity systems and technology development and investment of energy storage.

We constructed a transparent Macro-Energy Model (see Transparent Methods) to simulate the optimal design and operation of an idealized electricity system that consists of only VRE resources (wind turbine and solar photovoltaics (PV)) and energy storage (herein denoted as “VRE/storage system”) for the CONUS. The model simultaneously optimizes the planning decisions (how much capacity for any technology should be built) and the operating decisions (how much of each technology's deployed capacity should be dispatched) for VRE and storage assets, based on hourly VRE resource availability and hourly CONUS electricity demand, subject to first-principles constraints governing the electricity system. One of the critical system constraints that we evaluate is hourly reliability, or resource adequacy, for which there are at present specific regulatory requirements (ReliabilityFirst Corporation (RFC), 2017), as opposed to outages that result from infrastructure or operational failures that would result in additional loss of load hours and thus additional degradation of system reliability.

Historical hourly wind and solar generation potential between 1980 and 2015 is obtained from a reanalysis dataset that used a variety of data sources to produce a best-fit description of the state of the atmosphere every hour, with ∼60 km resolution across the CONUS (Shaner et al., 2018). We assumed exogenously weighted-average generation potential for both wind and solar energy (i.e., hourly 80-m wind and hourly downwelling surface solar radiation) (Shaner et al., 2018). The wind turbine's power curve is calculated as a cubic function of wind speed if the wind speed is between 3 and 15 m/s and a cubic function of the rated 15 m/s speed if the wind speed is between 15 and 25 m/s (Shaner et al., 2018). The average capacity factors for wind and solar generation assets were 0.38 and 0.22 for the 36 years considered. Hourly electricity demand data between July 2015 and July 2016 for the U.S. were obtained from the U.S. Energy Information Administration (EIA) , and replicated for all years evaluated in the simulations (Shaner et al., 2018; U.S. Energy Information Administration (EIA), 2017). The extended time series (36 years) allows hourly assessment of resource adequacy, while explicitly considering seasonal and interannual variability and infrequent weather-related events over a multi-decadal timescale that is commensurate with a typical lifetime of capital assets on an electricity grid.

To generalize the findings, we model generic energy storage characterized by capital cost, round-trip efficiency (90%), and energy loss over time (see Transparent Methods). We assumed a power-to-energy ratio of 1:1, and energy-related capacity and power-related capacity for energy storage are modeled together. Emerging energy storage technologies, such as flow batteries, and power-to-gas technologies, might have a much higher power cost but potentially a lower energy cost. The impact of power-limited energy storage is beyond the scope and not the central focus of this work.

In the main text, we report the least-cost optimization results for the baseline VRE/storage scenario ($1,500/kW wind turbine, $1,500/kW solar PV, and energy storage for 100% resource adequacy) (National Renewable Energy Laboratory (NREL), 2018; U.S. Energy Information Administration (EIA), 2018b). The Supplemental Information presents the least-cost optimization results for additional scenarios, including (1) wind/storage, (2) solar/storage, (3) VRE/storage with $1500/kW wind turbine and $750/kW solar PV, (4) VRE/storage with $750/kW wind turbine and $750/kW solar PV, (5) VRE/storage and dispatchable generators, as well as (6) the baseline VRE/storage system for resource adequacy of 99.97%. Furthermore, we parametrically varied the energy storage capital cost from $1,000 to $0.1/kWh. Altogether, we performed a total of 1,512 optimizations (36 years ∗ 6 storage costs ∗ 7 technology and resource adequacy scenarios).

Our idealized system is a closed electricity system, and we did not consider flexibility mechanisms associated with the conversion of electricity into heat, fuel, or other types of energy services. A (best-case) perfectly efficient transmission network was assumed to connect wind turbines and solar PVs, as well as load, across the CONUS. Real-world transmission losses and regionalization of generation and demand, by states or regional interconnection boundaries, would result in increased gaps between supply and demand relative to our idealized consideration of the CONUS as a single VRE generation area and load-balancing region. We assumed that the electricity system was constructed virtually instantaneously, and therefore did not consider decreases in asset cost due to technology learning . Nor did we consider switching costs associated with a transition from a legacy electricity system, or lock-in costs associated with a path-dependent electricity system transition designed to reduce and ultimately eliminate carbon emissions.

## Results

### System-Level Impacts of Low-Cost Energy Storage

Least-cost VRE/storage systems were evaluated across four orders of magnitude, from $1,000 to $0.1/kWh, of energy storage costs. Although previous studies have analyzed the role of storage costs in power systems, the computational complexity of more detailed and realistic models have limited the number of cases analyzed. The simplifying assumptions made by our idealized model might overestimate the resource adequacy of electricity systems but thus afford a longer-term and more comprehensive analysis of those systems.

Our analysis indicats that low-cost energy storage would have four critical system-level effects: (1) a decrease in total systems costs and mean electricity costs, (2) a change in the relative fractions of wind and solar electricity generation in least-cost systems, (3) a change in the roles that energy storage would play in least-cost systems, and (4) a reduction in the variability of hourly electricity costs.

#### Low-Cost Energy Storage Leads to Lower Electricity Costs

**Figure 1:**
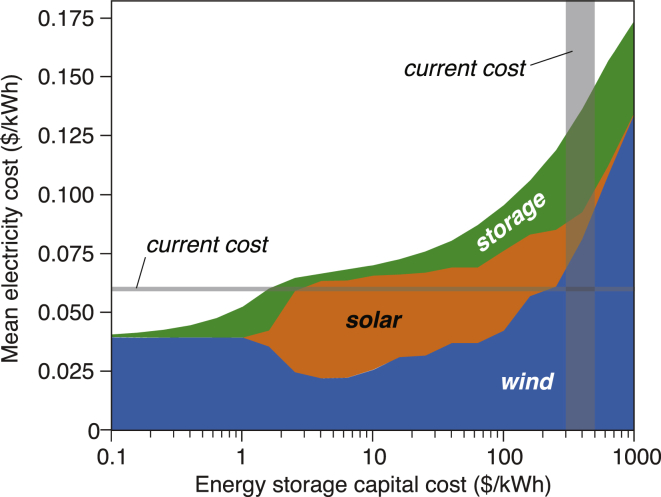
Effect of Storage Cost on the Mean Electricity Cost of the Least-Cost VRE/Storage Systems The optimization results shown are evaluated using hourly weather data and actual electricity demand for the year 2015. Current mean electricity costs ($0.06/kWh for the portion of generation cost in the annual average retail price of electricity) (U.S. Energy Information Administration (EIA), 2018c) and the range of current energy storage costs ($200–500/kWh) (Davis et al., 2018; Schmidt et al., 2019) are marked on the figure. Mean electricity costs for other VRE/storage systems (e.g., wind/storage, solar/storage, wind/solar/storage/dispatchable generation) and different VRE capital costs are available in Figure S5.

Figure 1 shows the mean cost of delivered electricity as a function of energy storage costs for the least-cost VRE/storage systems satisfying a 100% resource adequacy constraint. The least-cost VRE/storage system with $1,000/kWh storage results in mean electricity costs as high as $0.174/kWh, for the assumed VRE capital costs. For comparison, near-free (∼$1/kWh) storage costs would produce a 3-fold decrease in delivered electricity costs, to $0.052/kWh for the least-cost VRE/storage system.

**Figure 2:**
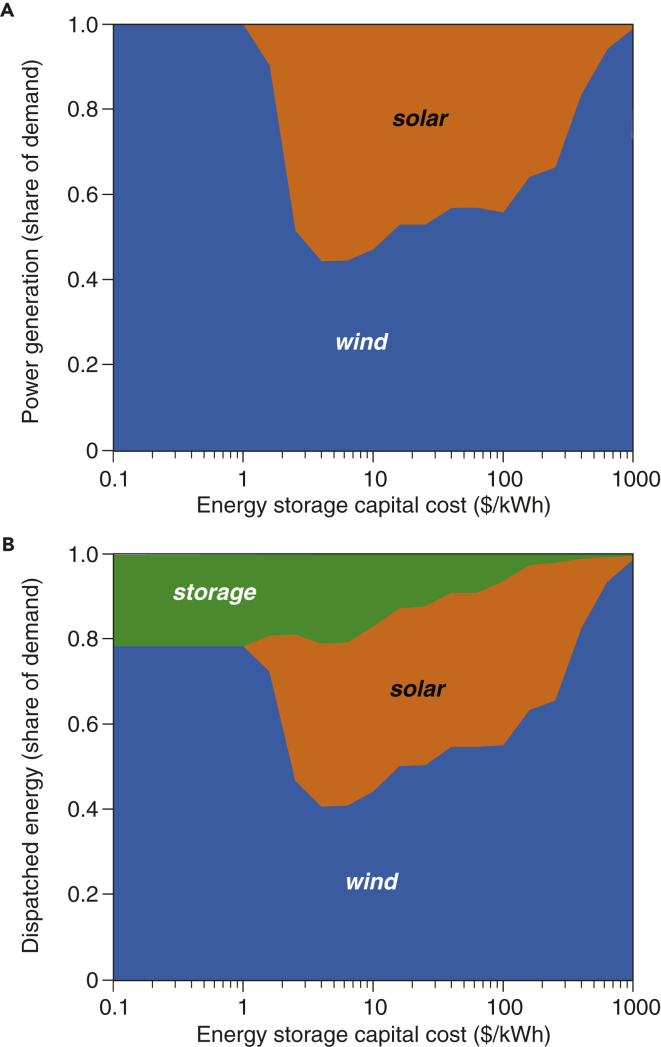
Effect of Storage Cost on Relative Shares of Net Electricity Generation and Dispatched Energy in the Least-Cost VRE/Storage Systems Curtailed generation of wind and solar electricity is excluded. The optimization results shown are evaluated based on hourly weather data and actual electricity demand for the year 2015. Daily-average least-cost dispatch mix is available in Figure S1. Results of wind and solar generation capacity, dispatched generation, and curtailed electricity for VRE/storage systems (e.g., wind/storage, solar/storage, wind/solar/storage/dispatchable generation) and different VRE capital costs are available in Figures S7–S10 and Table S3. As storage costs decrease from $1,000 to $1/kWh (from the right side toward the left), the relative share of solar generation would increase (A). A further decrease in storage cost would lead to the least-cost system utilizing wind generation exclusively with storage accounting for 22% of dispatched energy (B).

**Figure 3:**
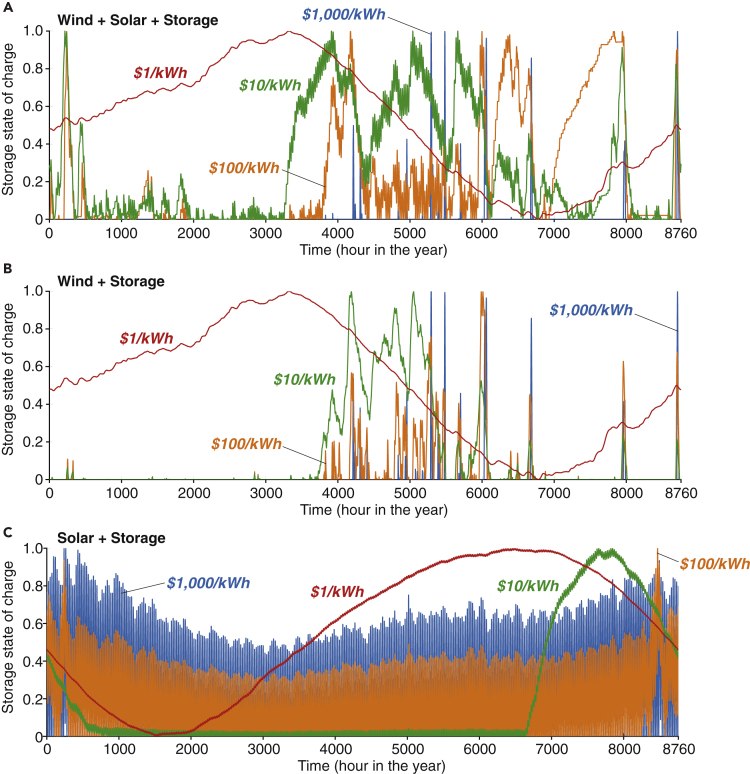
Effect of Storage Cost on the State of Charge (SOC) for Energy Storage in the Least-cost VRE/Storage Systems (A) wind/solar/storage system; (B) wind/storage system; (C) solar/storage system. The optimization results shown are evaluated using hourly weather data and actual electricity demand for the year 2015. Daily-average results of storage discharged and charged energy are available in Figures S1–S4. Storage SOC results for other VRE/storage systems (e.g., with dispatchable generation) and different VRE capital costs are available in Figure S11. As storage costs decrease from $1,000 to $1/kWh (from top to bottom), the role of storage changes from filling short-term gaps between VRE generation and hourly demand, to serving as seasonal storage for the VRE resources. The SOC profiles for energy storage are the same when energy storage costs are at or below $1/kWh for a given VRE/storage system . Note that the optimal storage capacity at different storage costs varies substantially (Figure 5A).

**Figure 4:**
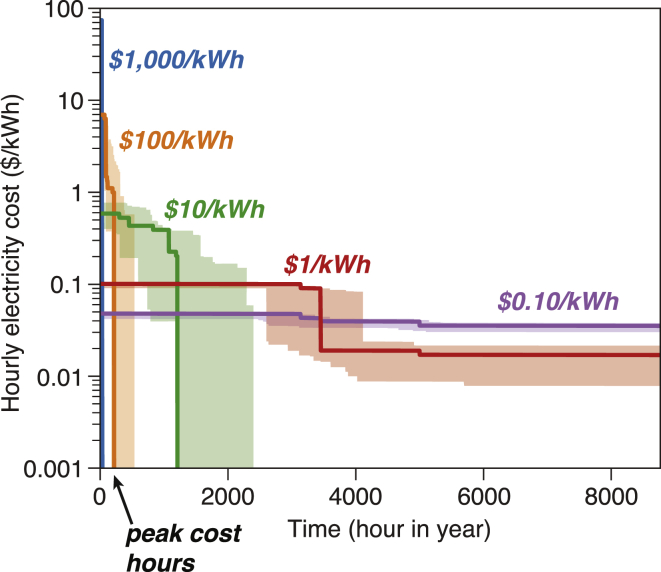
Effect of Storage Cost on the Cost Duration Curve for the Least-Cost VRE/Storage Systems Hourly electricity cost is calculated using the Lagrange dual variable of the hourly energy balance constraint for the VRE/storage system in the optimization model (Boyd and Vandenberghe, 2004) and represents the shadow price for the VRE/storage system to meet one additional unit of energy demand. We assumed a near-zero cost to represent operation and maintenance costs for wind, solar, and energy storage and a 90% round-trip efficiency for energy storage. These realistic representations lead to non-zero hourly costs. Bold lines show optimization results for the year 2015, and shaded areas show the range of results for independent annual optimizations (1980–2015). Results for other VRE/storage systems (e.g., wind/storage, solar/storage, wind/solar/storage/dispatchable generation) and different VRE capital costs are available in Figure S12. In VRE/storage systems with high storage costs (e.g., ≥$100/kWh, blue and orange curves), the hourly electricity costs at peak cost hours would also be high. At lower storage costs, the cost-duration curves would flatten out due to price arbitrage (green, red, and purple curves).

**Figure 5:**
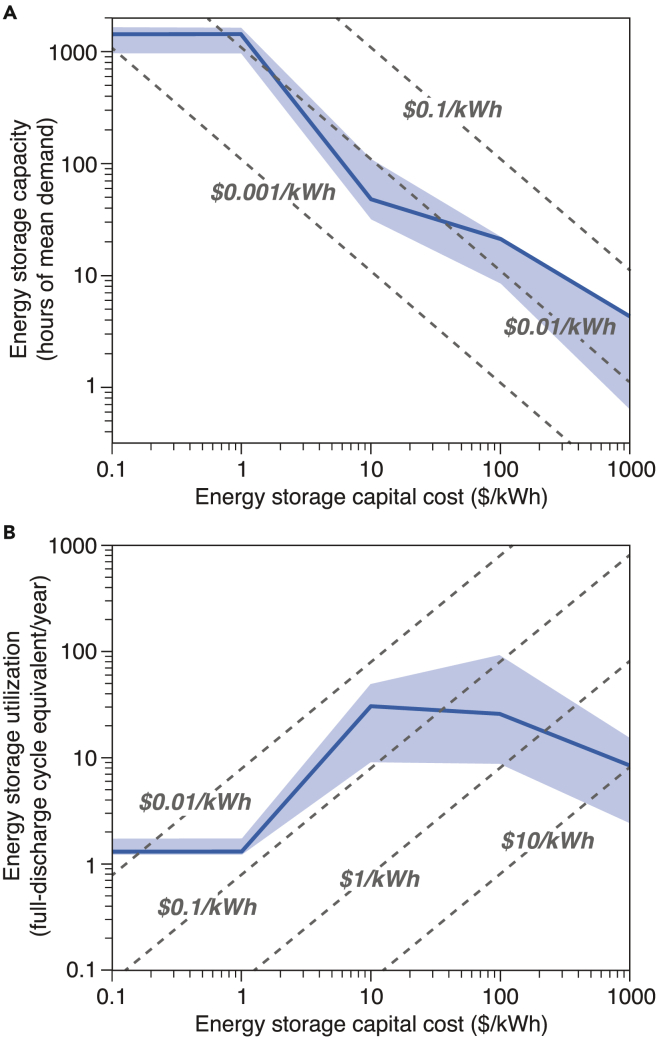
Effect of Storage Cost on the Deployed Storage Capacity and Utilization of Storage in the Least-Cost VRE/Storage Systems Bold lines show the optimization results for the year 2015, and shaded areas show the range of results from independent annual optimizations (1980–2015). Results for other VRE/storage systems (e.g., wind/storage, solar/storage, wind/solar/storage/dispatchable generation) and different VRE capital costs are available in Figures S13 and S14. If storage costs were to decrease, the capacity of storage deployed would increase (A), but expenditures on storage averaged over total delivered electricity by the VRE/storage system(dashed lines in A) would remain close to $0.01/kWh (roughly $39 billion per year for a U.S.-size system). In the meantime, utilization of storage would decrease (B), and the levelized cost of storage (the mean cost of electricity discharged from storage; dashed lines in B) would decrease from $10 to $0.01/kWh. Low-cost energy storage could also mitigate the impact of interannual VRE resource variability on storage capacity and utilization in the least-cost systems.

**Figure 6:**
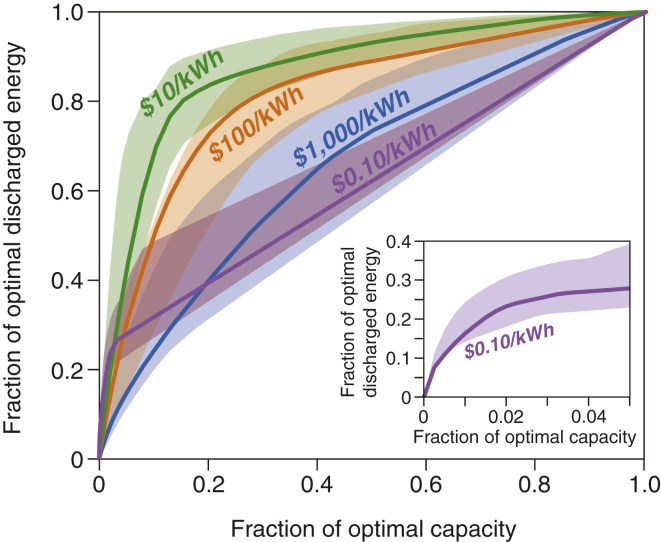
Relationships of Storage Discharged Energy and Storage Capacity at Different Storage Costs Bold lines show optimization results for the year 2015, and shaded areas show the ranges of results of independent annual optimizations (1980–2015). The plot for $1/kWh storage is identical to that for $0.1/kWh storage (purple). The inset figure shows the enlarged $0.1/kWh (purple) curve for a small fraction (0–0.05) of the optimal capacity. This figure is based on a reanalysis of the optimized results of storage capacity and utilization in the least-cost VRE/storage systems (Transparent Methods). Results for other VRE/storage systems (e.g., wind/storage, solar/storage, wind/solar/storage/dispatchable generation) and different VRE capital costs are available in Figures S15 and S16. Across a wide range of storage costs ($10–1000/kWh), energy storage follows the Pareto Principle: a small fraction of the capacity is well-utilized and the rest of the capacity is under-utilized. For example, in a VRE/storage system with $10/kWh storage, ∼20% of the storage capacity would supply more than 80% of the total discharged energy.

**Figure 7:**
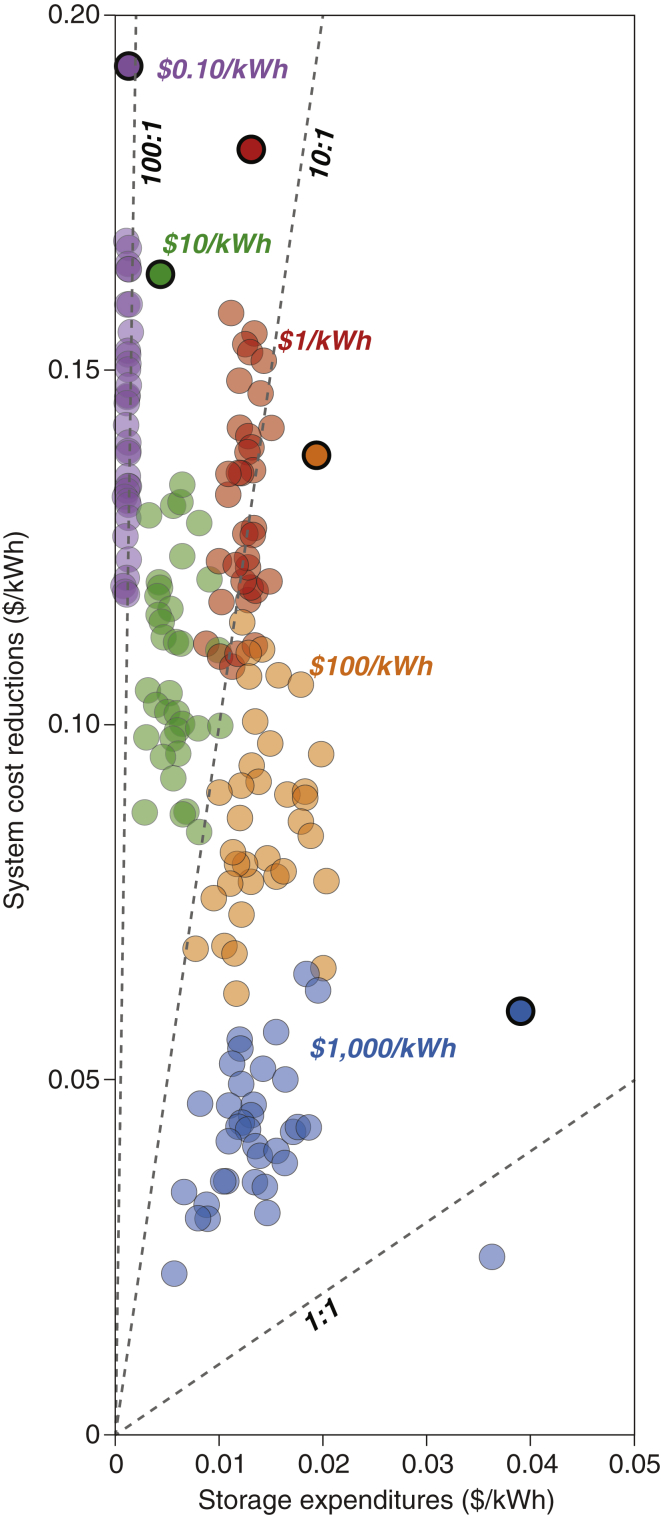
Relationships of System Cost Reductions and Energy Storage Expenditures for the Least-Cost VRE/Storage Systems at Different Storage Costs Each point represents the results of an independent annual optimization (1980–2015) for an assumed energy storage cost. Points with bold black border lines indicate the results for the year 2015. Results for other VRE/storage systems (e.g., wind/storage, solar/storage, wind/solar/storage/dispatchable generation) and different VRE capital costs are available in Figures S17 and S18. System cost reductions (defined as the difference in mean electricity cost for the least-cost system without and with energy storage) are consistently greater than expenditures on energy storage (averaged over total delivered electricity by the VRE/storage system) across a wide range of storage costs (denoted by points in different colors).

Near-free energy storage would eliminate the need to overbuild (by ∼340%) variable renewable electricity generation assets (i.e., curtailment of VRE generation) to achieve 100% resource adequacy. In contrast, the least-cost VRE/storage system with high-cost ($1,000/kWh) energy storage would require overbuilding these assets by >3-fold to generate enough renewable electricity to meet demand during periods of low wind and solar generation in a year (Figure S7). As shown in Figure S10, lower-cost energy storage substantially reduces the curtailment of renewable electricity. The amount of curtailment does not change gradually because, in least-cost systems, the generation mix and storage capacity also change in response to changes in storage costs (Table S3 and Figure 1, Figure 2, Figure 3, Figure 4, Figure 5, Figure 6, Figure 7and S7). For context, for the least-cost VRE/storage systems studied, near-free energy (∼$1/kWh) storage would result in a system cost savings of $472 billion per year (a product of $0.121/kWh and U.S. annual electricity demand of 3.9 × 10^12^ kWh per year) relative to analogous VRE/storage systems that instead used high-cost ($1,000/kWh) storage.

Figure 1 also shows that the delivered electricity costs in idealized VRE/storage electricity systems would depend nonlinearly on storage costs. Over the full range of storage costs considered, the largest reduction in system costs (from $0.174 to $0.095/kWh) would occur between storage costs of $1,000 and $100/kWh. Below $100/kWh, the same fraction (45%) of reduction in system costs would require energy storage costs to further decrease by an additional factor of 100. For perspective, current energy storage costs are in the range of $200–500/kWh (estimates differ by data source, system scope, and region) (Davis et al., 2018; Schmidt et al., 2019, 2017; U.S. Energy Information Administration (EIA), 2018a; Zakeri and Syri, 2015).

The nonlinear relationship between the mean electricity cost of the VRE/storage systems and energy storage capital cost is a result of the different mixes of VRE resources (see Figure 2 and the associated discussions) and distinct roles of energy storage (see Figure 3 and the associated discussions) in the least-cost VRE/storage systems optimized at different storage costs. Without energy storage, to ensure 100% resource adequacy, the system contained three times more wind and solar capacity than needed for meeting the average demand. Thus, the addition of energy storage replaces wind and solar capacity that would otherwise be needed for infrequent events, leading to sizeable overall system cost reductions. At low energy storage costs (below $100/kWh), large energy storage capacity is already in the optimized VRE/storage capacity mix. In this case, the variability in the hourly electricity prices becomes smaller, leaving a much tighter space for energy storage to perform price arbitrage. Furthermore, the optimized capacity mix of wind and solar generation is not constant at different energy storage costs, which in turn affects the value of energy storage for the whole system.

#### Low-Cost Energy Storage Shifts the Balance of Wind and Solar Generation in the Least-Cost VRE/Storage Systems

For either high-cost (∼$1,000/kWh) or near-free (∼$1/kWh) energy storage, the least-cost VRE/storage system would have a high ratio of wind electricity generation relative to solar electricity generation (Figures 2A, and S1). In these limiting cases, the variability of the renewable energy resource would be mitigated either by building substantially larger renewable energy capacity (and extensive curtailment in periods of low demand and high wind, for the case of high-cost storage) or by near-free energy storage. The balance between wind energy and solar energy at high-cost or near-free storage is driven by the different variability structures of wind energy (irregular over different time scales) and solar energy (consistent diurnal cycles and seasonal variations) as well as by the relative costs of wind energy and solar energy. At high storage costs, if wind energy has a lower levelized cost of electricity (LCOE) than solar energy (which is the case for the baseline scenario due to the higher capacity factor of wind energy and the same assumed capital cost, for simplicity, for both wind and solar generators), the least-cost VRE/storage system would predominately deploy and dispatch wind energy because building out solar capacity does not help with times of peak mismatches between supply and demand (e.g., evenings). At very low storage costs, wind energy predominates in the baseline scenario because of lower LCOE (again, owing to the higher capacity factor of wind in conjunction with the same assumed capital costs for wind and solar generators). If solar energy has a lower LCOE than wind (in the alternative scenario where we assumed $750/kW solar PVs and $1,500/kW wind turbines), wind and solar energy would complement each other in the optimized systems. The ratio of net electricity generation between wind energy and solar energy is 4:1 for costly storage and 3:7 for near-free storage (Figures S3 and S8).

In contrast, for intermediate energy storage costs ($10–1,000/kWh), a generation mix of both wind and solar electricity would be present in the least-cost VRE/storage system (Figure 2A and S1). For example, with $10/kWh energy storage, solar PV would provide approximately the same electricity generation as wind turbines (excluding curtailment). In this case, energy storage would primarily smooth out the diurnal cycle for solar PV and thus increase the value of these assets (Figure 3A).

For >$150/kWh energy storage, the share of electricity discharged from storage assets in the least-cost VRE/storage systems remains small (<3%) but would be essential in certain hours (Figures 2B and 3A). In contrast, with near-free energy storage, up to 22% of electricity demand in the least-cost system would be met by storage, with the remaining demand met directly by VRE generation (Figure 2B). In other words, 80% of electricity demand could be met by wind and solar resources without substantially overbuilding VRE generation capacity. This finding is consistent with previous studies that used more detailed and complex engineering models of the electric power grid (MacDonald et al., 2016) as well as those that evaluated geophysical characteristics of wind and solar resources over the CONUS (Shaner et al., 2018).

#### Energy Storage Performs Distinct Roles in the Least-Cost VRE/Storage Systems Optimized at Different Storage Costs

The role of energy storage changes from high-cost storage competing with curtailment to fill short-term gaps between VRE generation and hourly demand to near-free storage serving as seasonal storage for VRE resources. In our optimized VRE/storage system, this transition is driven by deep reductions in energy storage costs. However, in systems that already have substantial VRE capacity deployment, this transition might be driven by the need to reduce curtailed electricity or price arbitrage.

Figure 3 shows the state-of-charge (SOC) for energy storage in the least-cost VRE/storage systems at different technology assumptions and energy storage costs. A reduction in the SOC for storage represents energy discharged from storage to meet electricity demand, and indicates when and how much energy storage would be needed to ensure adequacy of VRE resources.

At high costs (>$100/kWh), energy storage is charged and discharged within a day. This could happen irregularly for wind-only or wind-dominated (e.g., baseline) systems (Figures 3A and 3B) but takes place daily for solar-only systems to smooth out the diurnal solar irradiation cycle (Figure 3C). At costs between $1 and $100/kWh, the operational cycle of energy storage extends from a day to several days or even weeks, which is more salient for wind-only or wind-dominated systems (Figure 3). At near-free costs, energy storage cycles on an annual basis to balance out VRE generation and electricity demand for any mix of VRE sources considered (Figure 3). In this case, energy storage discharges to meet the annual generation trough of the VRE resources in late spring and summer (mid-May to mid-September) for wind-only or wind-dominated systems, or fall and winter (mid-October to February) for solar-only or solar-dominated systems.

#### Energy Storage Reduces Variability in Hourly Electricity Costs in the Least-Cost VRE/Storage Systems

Figure 4 shows the cost-duration curve for the least-cost VRE/storage systems. A cost-duration curve is obtained by sorting hourly electricity costs from high to low in a given year. Hourly electricity cost is calculated using the Lagrange dual variable of the hourly energy balance constraint for the VRE/storage system in the optimization model (Boyd and Vandenberghe, 2004), and represents the shadow price for the VRE/storage system to meet one additional unit of energy demand.

At high storage costs ($1,000/kWh; blue curve in Figure 4), hourly electricity costs would be unevenly distributed: for 99% of the time, hourly electricity costs would be close to zero because of the abundance of VRE generation capacity to meet the additional electricity demand for these hours; however, for 19 h in a year, the hourly marginal electricity costs would reach $73/kWh (for the assumed VRE/storage system). These high hourly costs are a consequence of building VRE capacity that would be used for only a few hours per year. The high mean electricity costs (Figure 1) are a direct result of these few but highly expensive hours (“peak cost hours”; Figure 4).

Low-cost energy storage would greatly reduce variability in the hourly electricity cost in the least-cost VRE/storage systems (Figure 4). The corresponding cost duration curves would become flatter (e.g., red and purple curves in Figure 4) as energy storage costs decrease. An order-of-magnitude reduction in storage costs (in the range from $1,000 to $0.1/kWh) would lead to an order-of-magnitude reduction in hourly electricity costs for peak cost hours, by avoiding additional VRE capacity that otherwise would be needed only for these peak cost hours. In contrast, owing to price arbitrage, in these VRE/storage systems, utilization of energy storage would increase hourly electricity costs slightly for the non-peak-cost hours. Nevertheless, cost reductions for peak cost hours substantially outweigh cost increases for non-peak-cost hours, leading to overall system cost reductions as energy storage costs decline.

### Economic Challenges for Grid-Scale Energy Storage

The levelized cost of storage (LCOS) is a widely used metric in the techno-economic assessment of energy storage technologies (Davis et al., 2018). As defined in Transparent Methods (Equation 3), LCOS is the ratio of the annualized capital cost of energy storage divided by the total discharged electricity from energy storage. In other words, LCOS represents the levelized cost of the discharged electricity from energy storage if charging were free; it also represents the levelized arbitrage spread needed to recover fixed costs of cycling the storage per unit of energy discharged.

As shown in Figure 5A, in the least-cost VRE/storage systems, lower-cost energy storage would lead to an increased deployment of storage, but at the expense of reduced storage utilization. If energy storage costs were to decrease from $1,000 to $0.1/kWh, the optimal capacity of storage deployed, represented in terms of the mean hourly electricity demand met by a full discharge of the stored energy, would increase from 4 to 1,437 h (about 2 months), and contribute a fixed cost of ∼ $0.01/kWh to the least-cost VRE/storage system (Figure 5A). For a system sized to meet the current U.S. electricity demand of about 3.9×10^12^ kWh per year, the expenditure on energy storage would be roughly $39 billion per year.

The relationship between system expenditure on storage and cost of storage depends on the relative costs and resource availability of wind and solar energy. In a wind-dominated VRE system (i.e., wind has a lower LCOE than solar; Figure 5A), the system expenditure on storage is relatively constant across a wide range of energy storage costs. For a solar-dominated VRE system (i.e., solar has a lower LCOE than wind; Figure S13), the system expenditure on storage reduces as energy storage cost declines. Specifically, the investment needed for near-free ($0.1/kWh) energy storage would be two orders of magnitude lower than that for high-cost ($1,000/kWh) energy storage (Figure S13). This behavior occurs because solar and storage can provide dispatchable generation when paired at specific capacity ratios. Thus, in terms of storage investment, the increase in storage capacity is outweighed by the reduction in storage costs in a solar-dominated VRE/storage system.

Driven by reduced storage cost, the utilization of energy storage would first increase from 9 cycles (full discharge equivalent) per year to 31 cycles (full discharge equivalent) per year, and would then decrease to 1 cycle (full discharge equivalent) per year for the baseline VRE/storage system (Figure 5B; see also Figure 3), and the LCOS of energy storage would decrease from $9.22/kWh to $0.01/kWh (Figure 5B). Because the capital cost of energy storage is proportional to the product of LCOS and utilization of energy storage (Equation 3 in Transparent Methods), these results imply that at least three orders-of-magnitude reductions in storage costs from current values would be required for the least-cost VRE/storage systems to produce 100% reliable electricity without substantially overbuilding VRE capacity.

In the least-cost VRE/storage systems, energy storage roughly follows the Pareto Principle, as 20% of the storage capacity supplies 80% of the discharged energy, whereas the additional 80% of the storage capacity supplies only the remaining 20% of discharged energy (Figure 6; algorithm available in Transparent Methods). With increasing capacity, additional storage is used less frequently. When storage is cheaper than building additional VRE generation capacity, the least-cost VRE/storage system would deploy and operate storage to fill the gaps between VRE generation and electricity demand. The need for the discharged energy from storage assets is thus not constant in frequency or quantity (Figure 3). Energy storage sized to meet the maximum of such needs is bound to be underutilized most of the time because “the maximum need” happens rarely (Figure 3). Moreover, low-cost energy storage would decrease the installed VRE capacity (by reducing curtailment) and increase the energy storage capacity, further exacerbating the low utilization of energy storage.

As discussed above, energy storage performs distinct roles in the least-cost VRE/storage system optimized at different storage costs (Figure 3). In Figure 6, the curves for $1 and $0.1/kWh storage show distinctly different shapes compared to those for other storage costs. At these near-free costs, with orders-of-magnitude larger capacity deployed (Figure 5A), energy storage serves primarily as seasonal storage for the VRE resources (Figure 3), thus exhibiting a linear pattern in Figure 6. Only a small fraction of the storage capacity (which is comparable with the optimal energy storage capacity at higherstorage costs) exhibits a concave pattern following the Pareto Principle (Figure 6, inset figure).

### System Benefits of Grid-Scale Energy Storage

Figure 7 shows the system cost reductions that would be provided by energy storage relative to expenditures on energy storage for the least-cost VRE/storage systems optimized at storage costs ranging from $1,000 to $0.1/kWh. The system cost reduction is calculated as the difference in the mean electricity cost of the least-cost VRE system (i.e., without storage) and that of the least-cost VRE/storage system.

Low-cost energy storage would lead to substantial system cost reductions (Figure 7; also consistent with Figure 1) for the least-cost VRE/storage systems. The ratio of system cost reductions to energy storage expenditures would increase substantially if energy storage costs were to decrease. For most years studied, $1,000/kWh energy storage would produce a system-cost-reductions-to-storage-expenditures ratio of less than six, whereas $10/kWh energy storage would lead to system cost reductions that are more than 10 times greater than energy storage expenditures. Consequently, reductions in energy storage cost may not alone expand the size of the grid-scale energy storage market (Figure 5A), but instead would primarily promote the public good by delivering electricity cost reductions for end users. These conclusions could differ for other energy storage markets, such as electric vehicles in the transportation sector.

### Sensitivity to the Variability of Renewable Energy Resources

The interannual variability of VRE resources could have a substantial impact on the optimal design and operation of electricity systems based primarily on variable renewable generation assets. For the least-cost VRE/storage systems at high storage costs ($1,000/kWh), the evaluated metrics (Figure 4 cost-duration curves; Figure 5 storage capacity and utilization; Figure 7 system cost reductions due to energy storage) showed substantial variations across 36 independent annual optimizations, based on resource availability for years 1980–2015. In contrast, at lower storage costs, these studied metrics started to converge across the least-cost VRE/storage systems optimized for different years. Hence another system benefit of ultralow-cost energy storage is associated with the flexibility to mitigate interannual variability of wind and solar electricity generation. This flexibility would be especially valuable in light of the profound uncertainty in energy systems planning that arises from socioeconomic activities, technology innovation, development, and adoption, and climate change (Sherwin et al., 2018).

### Sensitivity to Renewable Energy Technology Assumptions

In Supplemental Information (Figures S1–S18), we present results for different specifications of VRE/storage systems (e.g., wind/solar/storage, wind/storage, and solar/storage) and different VRE costs (baseline wind energy costs, and 50% lower solar energy costs; 50% lower capital costs for both wind energy and solar energy). Across these scenarios, the least-cost VRE/storage systems are governed by a fundamental trade-off between building additional VRE capacity and deploying and utilizing storage. At lower VRE costs, the least-cost VRE/storage systems would avoid electricity curtailment from additional VRE capacity only if energy storage costs further reduce to $0.1/kWh, an order of magnitude lower than the baseline case. This is because lower VRE costs make curtailment less costly to the VRE/storage systems ceteris paribus, so energy storage has to be even cheaper to compete with overbuilding VRE capacity.

The system cost reductions due to low-cost energy storage depend on technology cost and technology availability assumptions. In solar-only VRE/storage systems, energy storage leads to >2*x* system cost reductions than the baseline case (with both wind and solar) for the same storage cost (Figures S5 and S17). Furthermore, system cost reductions for wind-only VRE/storage systems are much more sensitive to weather variations over 1980–2015 than the solar-only VRE/storage systems (Figure S17).

We caution that specific system costs are sensitive to changes in asset costs. For instance, a system with 50% lower VRE capacity costs and $1,000/kWh storage would produce 100% reliable electricity at a cost ($0.097/kWh) comparable with a system with the baseline VRE capacity costs and $100/kWh storage ($0.095/kWh). Hence a more meaningful comparison is associated with relative cost changes between systems evaluated in this work. These relative system cost comparisons provide a tool to uncover the fundamental relationships between VRE technologies and storage for the least-cost VRE/storage systems. Reductions in generation asset costs in the future would lower system costs. However, the parameterized ratios between generation costs and energy storage costs would still apply, so decreases in future wind/solar generation costs would require proportionate reductions in energy storage costs for storage to be deployed relative to curtailment of wind and solar generation in least-cost reliable electricity systems.

### Sensitivity to Dispatchable Generators

Previous studies (Sepulveda et al., 2018) showed that dispatchable low-carbon generation technologies consistently lower decarbonized electricity system costs. Our results (Figure S5) support this conclusion when dispatchable generators (modeled after natural gas combined cycle power plants with carbon capture and storage technology) were included in the VRE/storage electricity system. In this case, dispatchable generators generated about 60%, 50%, and 20% of electricity demand when the capital costs of energy storage were $1000/kWh, $100/kWh, and $10/kWh, respectively (Figures S4 and S8).

As a result, system cost reductions due to declining energy storage costs in the VRE/storage/dispatchable generation systems shrink substantially compared to the baseline VRE/storage systems (without dispatchable generation) for the same energy storage cost. At storage costs of $10–1,000/kWh, energy storage leads to only about 10% of the system cost reductions when dispatchable generation is present compared with the baseline case without dispatchable generation (Figures S5 and S18). However, system cost reductions caused by low-cost storage still outweigh the expenditure on storage even when dispatchable generation is present (Figure S18).

The main conclusion that energy storage cost would have to be several orders of magnitude lower than today's cost for the least-cost VRE/storage systems to generate reliable electricity without overbuilding VRE capacity is still valid. Indeed, when dispatchable generation is present, storage cost would have to be even lower than the baseline case (to $0.1/kWh) for the VRE/storage system to eliminate dispatchable generation (Figures S4, S5, and S7).

### Sensitivity to Resource Adequacy Requirements

In Supplemental Information (Figures S19–S29 and Tables S4 and S5), we present results for the least-cost VRE/storage systems optimized for a resource adequacy constraint of 99.97% rather than 100% over a year. The mean cost of electricity for the 99.97% case would be lower than that for the 100% case (Table S5). With $1,000/kWh storage, the least-cost capacity mix of VRE and storage for the 99.97% case is different from that for the 100% case. Compared with the case of 100% resource adequacy, 13 times more solar capacity would be built (18 GW versus 260 GW), but wind capacity and energy storage capacity would decrease by 11% (4,402 GW versus 3,932 GW) and 38% (1,921 GWh versus 1,190 GWh) (Tables S4 and S5). As a consequence, the mean electricity cost is 13% cheaper. When storage costs less than $100/kWh, the optimal capacity mix for these two cases of resource adequacy requirements would be similar (Table S5).

## Discussion

### Roles and Requirements of Energy Storage for Variable Renewable Energy

Geophysical constraints on the variability of wind and solar resources are a substantial driver of system costs owing to the need to oversize VRE capacities or deploy adequate storage to avoid infrequent, long-duration outages as well as compensate for seasonal resource variability. Even with the favorable assumptions that underpin the idealized VRE/storage systems modeled herein, specifically lossless transmission and ideal utilization of the solar resource simultaneously over four time zones, as well as instantaneous aggregation of the wind resource over the entire CONUS, substantial gaps can nevertheless be present between hourly electricity demand and VRE generation over a representative year (Figure 2 in Shaner et al. (2018)).

The results indicate that energy storage faces “double penalties” in VRE/storage systems: with increasing capacity, (1) the additional storage is used less frequently and (2) hourly electricity costs would become less volatile, thus reducing price arbitrage opportunities for the additional storage. Furthermore, the role of energy storage changes from high-cost storage competing with curtailment to fill short-term gaps between VRE generation and hourly demand to near-free storage serving as seasonal storage for VRE resources.

The economics of energy storage depends critically on how storage is used (Davis et al., 2018; Li et al., 2017; Schmidt et al., 2019; Zakeri and Syri, 2015). For instance, Davis et al. estimated that the cost of discharged electricity from the same lithium-ion batteries could rise from $0.14/kWh to $0.50/kWh if the use of storage were to decrease from daily cycling to weekly cycling (Davis et al., 2018). Hence storage is inherently expensive for applications in which large capacity of energy storage is infrequently used, as we have shown in the studied VRE/storage systems. To avoid overbuilding VRE capacity and curtailment of VRE electricity, energy storage should have adequate capacity to serve as seasonal storage for the VRE resources. An ultralow storage cost is thus required to make storage economically competitive either with the curtailment of VRE capacity or with other mechanisms that provide flexibility to mitigate the variability of VRE assets while maintaining system resource adequacy.

The general conclusion is that the needed cost reductions in energy storage in a reliable variable renewable energy (VRE) electricity system are not factors of 2–5 from present values, as might be deduced from an LCOE analysis, but are on the order of 100-fold relative to current capacity costs for grid-scale energy storage (such as batteries). This 100-fold required reduction is driven by the fundamental trade-off in system design between overbuilding VRE capacity and building and utilization of storage to mitigate short-term and long-term weather-related variability for VRE resources, even assuming perfect transmission of wind and solar generation aggregated over the CONUS to all load. This finding is robust for different VRE costs and systems with dispatchable generators (such as natural gas, hydropower, geothermal, and nuclear fission). Moreover, if VRE costs were to decrease, then storage costs would have to be even lower to favor storage relative to VRE capacity.

Our results are in qualitative accord with and are reinforced by recently reported work (Ziegler et al., 2019) performed in parallel with our study that evaluated the least-cost combination of wind, solar, and energy storage assets to meet simplified yet artificial demand profiles using 20 years of renewable energy data with an hourly resolution at four locations across the United States and concluded that <$20/kWh storage energy capacity costs are necessary for “cost-competitive, reliable baseload electricity generation.” Similarly, through the study of a CONUS-scale VRE/storage system, our findings extend and quantify the conclusion from Safaei and Keith (2015) that seasonal energy storage is not economical at $100–200/kWh energy storage costs for a wind/storage system in Texas, U.S.. Our analysis of VRE/storage systems with and without dispatchable generators highlights the role of dispatchable generators in future low-carbon electricity systems (such as VRE/storage systems), and corroborates Sepulveda et al. (2018)'s finding about “firm” dispatchable low-carbon generation resources.

### Implications for Energy Storage Technology Development

The costs of electrochemical energy storage technologies (such as Li-ion, Ni-MH, Ni-Cd) have reduced substantially over the past several decades owing to public and private research and development (R&D) and economies of scale (Davis et al., 2018; Schmidt et al., 2017). Recent estimates show that grid-scale energy storage can cost as low as $250/kWh, although cost estimates vary by chemistry, manufacturer, and region (Davis et al., 2018; Schmidt et al., 2017; Zakeri and Syri, 2015). Continued R&D and expanded penetration in specific markets (e.g., personal electronics and, increasingly, personal automobiles) may further drive down storage costs, but near-free energy storage (∼$1/kWh) is unlikely to be attained in the near term. For context, the U.S. Department of Energy recently set an R&D target of $100/kWh for utility-scale energy storage (U.S. Department of Energy (DOE), 2013, U.S. Department of Energy (DOE), 2018). Costs far below $100/kWh are likely not feasible through economies of scale associated with mass manufacturing of any currently known battery technology, due to cost limitations associated with the raw materials required for energy storage itself (Schmidt et al., 2017). Our analysis thus underscores the need to research, demonstrate, and deploy fundamentally new battery chemistries for infrequently utilized energy storage applications that only utilize highly abundant, very inexpensive raw materials in conjunction with ultralow-cost manufacturing processes (Li et al., 2017).

The steep diminishing returns for energy storage cost reductions in VRE/storage systems indicate that other technologies that could provide flexibility, such as expanded electrification and demand response in heating, industry, and transportation; low energy-cost long-duration storage technologies (e.g., thermal storage, power-to-gas, and power-to-liquid fuels); and high-voltage direct-current (HVDC) transmission lines are likely to be cost-effective components for future zero-carbon energy systems. Furthermore, as has been previously noted (MacDonald et al., 2016; Mai et al., 2018; Sepulveda et al., 2018), the availability of dispatchable generation can markedly reduce the costs of electricity systems otherwise dominated by solar and wind power. Publicly funded R&D at both device and system levels could potentially improve the technical and economic performances of these technologies, and facilitate the integration and coordination of these technologies into reliable renewable energy systems.

In the least-cost VRE/storage systems considered herein, the amount of money spent on energy storage, for systems meeting current CONUS electricity demand, would remain mostly constant ($39 billion per year, which depends on the costs and capacity factors of VRE technologies) across a wide range of energy storage costs ($1–1,000/kWh). Hence, in these scenarios, storage innovations would provide investors with a competitive advantage, but would not substantially increase the overall market size for grid-scale storage. The total electricity system costs may decrease substantially as a result of declining storage costs, which may motivate public support for the R&D of energy storage technologies. The reductions in system costs (compared to the same electricity system but without energy storage) are almost always greater than the expenditure on energy storage for the range of storage costs studied ($1,000–0.1/kWh). Moreover, the ratio of system cost reductions to storage expenditures would increase substantially if energy storage costs were to decline from $1,000/kWh to $0.1/kWh. This potential mismatch between private and public benefits suggests a need for public R&D support for energy storage technologies to account for the positive externality and promote the public good.

Although we focused primarily on the impact of deep reductions in energy storage costs on highly reliable VRE/storage systems, we note that energy storage provides many other grid services such as capacity values and ancillary services (Castillo and Gayme, 2014; U.S. Department of Energy (DOE), 2013, U.S. Department of Energy (DOE), 2018). Future research may assess how to best design and operate energy storage technologies for these applications, develop new methods and tools to quantify multiple values of energy storage simultaneously, and study electricity market designs that efficiently manage variable renewable energy resources and energy storage (U.S. Department of Energy (DOE), 2013, U.S. Department of Energy (DOE), 2018).

### Benefits of the Macro-Energy Modeling Approach

The Macro-Energy Model provides a transparent approach to evaluate the degree to which energy storage costs would have to decrease to ensure resource adequacy in an electricity system with high shares of VRE generation. It assesses the fundamental system trade-off associated with building and operating energy storage and overbuilding VRE assets (and curtailment of VRE electricity) to ensure resource adequacy of the VRE/storage system with explicit consideration of weather-related short-term and long-term VRE variability.

The use of a macro-energy model has allowed us to evaluate 1,512 (36 years ∗ 6 storage costs ∗ 7 system designs) optimization cases to assess the impact of energy storage costs on VRE/storage systems with various levels of resource adequacy requirements in the context of short-term and long-term weather-related variability of VRE resources on a multi-decadal timescale. In comparison, it would be challenging to achieve these tasks using state-of-the-art power engineering models or energy system models that include refined geographical representations, high temporal resolutions (e.g., 5 min or shorter time steps), and sophisticated technical details. The role and utility of these emerging macro-energy systems models have been discussed recently in detail (Levi et al., 2019).

### Limitations of the Study

Although batteries and all storage technologies, in general, require specification of both energy and power costs, energy storage costs are a vital quantity to assess the role of energy storage in VRE/storage systems with respect to daily, seasonal, and interannual resource variability. Storage can provide additional services to a reliable grid, including 5-min load balancing and short-term stability services, for which power costs are an essential metric, but these services are beyond the scope of this work. The focus of this work is the fundamental trade-off between overbuilding VRE capacity (and thus curtailment of VRE generation) and building and utilization of storage to achieve the requisite hourly resource adequacy for the VRE/storage systems over a long time period. To cost-effectively obtain short-term and long-term resource adequacy and reliability would likely require the utilization of several types of storage technologies synergistically, each of which has individual advantageous characteristics concerning power costs or energy costs.

This work used a historical demand profile owing to the limited availability of modeled future demand data at hourly resolution (Mai et al., 2018). The US EIA electricity demand dataset (U.S. Energy Information Administration (EIA), 2017) was the only reasonably complete and reliable record available at the time when this study was conducted. Consequently, the US EIA demand data (between July 2015 and July 2016) were replicated for each of the years evaluated in this study, with correction for leap years where needed. This approach allows robust investigation of the roles of energy storage in VRE/storage systems with explicit consideration of daily, seasonal, and interannual variability in VRE resources. High solar output and high seasonal average demand might be correlated for the year 2015 owing to the impact of solar irradiance on cooling demand. The robustness of the findings was validated through single-year optimizations using hourly weather data for each year in 1980–2015, which contains substantial interannual variability in VRE resources. Future electricity demand profiles could be different from historical ones due to accelerated electrification of transportation, heating, and industry, which would occur alongside any transition to VRE/storage systems. Furthermore, the flexibility of these demands at different timescales and as a response to various incentives would lead to distinct roles for energy storage in such systems. The study of energy storage and flexibility of demand in a variety of possible future electricity systems is beyond the scope of this study.

Actual electricity systems are spatially segregated and linked by capacity-limited transmission lines. The impacts of VRE resources are likely to be amplified by congested transmission lines and various grid conditions, which would require a more detailed electricity system model to assess in full. A more sophisticated “real-world” model that accounts for transmission losses and regionalization of generation would exacerbate the gaps between VRE resources and electricity demand and thus further emphasize the need for energy storage to effectively mitigate the variability of VRE resources relative to curtailment or other means of flexibility. Use of a shorter load balancing time in modeling, such as 5 min, would be appropriate to simulate other grid stabilization and load balancing mechanisms (Arabali et al., 2014; Bahramirad et al., 2012; Xu and Singh, 2014; Zhang et al., 2011) but would not directly improve characterization of hourly averaged VRE resource variability and demand variability that fundamentally determine the behaviors of VRE/storage systems.

In this work, we optimized the VRE/storage system on an hourly basis for 1 year. To illustrate the impact of interannual variability of VRE resources, the analysis was repeated using hourly weather data for each year from 1980 to 2015. Owing to computational (memory) limitations associated with simultaneous optimization of hourly storage capacity over 5 years, we are unable to solve the optimization for VRE/storage systems with weather data for more than 5 years at a time. Furthermore, the assumption of perfect foresight in multi-decade planning is not realistic, and the forecast data would nevertheless contain errors, both of which are fundamental challenges for long-term planning. In the additional runs, multi-year optimizations led to a slightly higher mean electricity cost and larger energy storage capacity than those obtained from single-year optimizations. These results are driven by a higher degree of mismatch between VRE resources and electricity demand over multi-year periods than in a single year. The general conclusions of this work, arising from the system design trade-off in overbuilding VRE capacity and building and utilizing energy storage to address the mismatch between VRE resources and electricity demand at different timescales, would remain robust in multi-year or multi-decade optimization cases, but the exact numerical results could vary from those specified herein.

### Resource Availability

#### Lead Contact

Further information and requests for resources should be directly to and will be fulfilled by the Lead Contact, Ken Caldeira (kcaldeira@carnegiescience.edu).

#### Materials Availability

This study did not generate any new materials.

#### Data and Code Availability

Original data and processed results (including underlying data for all figures in the main text) have been deposited to Zenodo: 10.5281/zenodo.3995063. The code of the macro-energy model is available at https://github.com/carnegie/SEM_public/tree/Tong_et_al_2020.

## Methods

All methods can be found in the accompanying Transparent Methods supplemental file.

## Supplemental Information

Document S1. Transparent Methods, Box S1, Figures S1–S29, and Tables S1–S5

## Footnotes

Supplemental Information can be found online at https://doi.org/10.1016/j.isci.2020.101484.
