# Supplementary material for: Effects of Deep Reductions in Energy Storage Costs on Highly Reliable Wind and Solar Electricity Systems
Source: iScience. 2020 Aug 20;23(9):101484. doi: 10.1016/j.isci.2020.101484 (PMC7492991; doi:10.1016/j.isci.2020.101484)
Supplement: Document S1. Transparent Methods, Box S1, Figures S1–S29, and Tables S1–S5 [file mmc1.pdf]

iScience, Volume *23*

Supplemental Information

Effects of Deep Reductions in Energy Storage Costs on Highly Reliable Wind and Solar Electricity Systems

Fan Tong, Mengyao Yuan, Nathan S. Lewis, Steven J. Davis, and Ken Caldeira

Transparent Methods

Definitions

Annualized capital cost (ACC) is calculated by multiplying capital cost (CC) with the capital recovery rate (CRF) (1), which is then determined using the discount rate, *i*, and asset lifetime, *n* (2).

$$ACC = \gamma \times CC \tag{1}$$

$$\gamma = CRF = \frac{i \times (1+i)^n}{(1+i)^n - 1} \tag{2}$$

Levelized cost of storage (LCOS), defined in (3) below, encompasses three variables for energy storage: annualized capital cost, discharged energy, and deployed capacity:

$$levelized\ cost\ of\ storage\ (\mathrm{LCOS}) = \frac{ACC^{\mathrm{es}}}{full\text{-}discharge\ cycles\ per\ year} = ACC^{\mathrm{es}} \times \frac{C^{\mathrm{es}}}{\sum_{t=1}^{N} D_t^{es,\,discharge}} \tag{3}$$

where C^{es} represents deployed capacity of energy storage (kWh), and $D_t^{es,\,discharge}$ represents hourly discharged electricity from energy storage (kWh) for hour t. N is the total number of hours in a year. Note that for simplicity, we assumed zero operation and maintenance costs.

Macro-Energy Model

We use a simple macro-energy optimization model to investigate the influence of deep reductions in energy storage costs on the design and operations of variable renewable electricity systems. The model optimizes simultaneously the capacity deployment of power generation and storage assets as well as the dispatch order of deployed assets to minimize the total system cost. The model requires electricity demand to be met for every hour. The model assumes perfect foresight and is a deterministic linear programming optimization model. The model considers fundamental physical constraints such as energy balance between electricity generated and electricity consumed at any hour, energy balance for energy storage at any hour, and constraints linking dispatched generation and potential generation (determined by capacity) for each generation technology considered. The mathematical formulation of the optimization model is shown in (4)-(12):

$$\min_{C^w, C^s, C^{es}, D_t^w, D_t^s, D_t^{es,\,discharge}, D_t^{es,\,charge}} acc^w \times C^w + acc^s \times C^s + acc^{es} \times C^{es} + m \times D_t^{unmet} \tag{4}$$

$$s.t.\ D_t^w + D_t^s + D_t^{es,\,discharge} + D_t^{unmet} = d_t + D_t^{es,\,charge},\ t = 1,\ldots,n \tag{5}$$

$$D_t^w \le cf_t^{\,w} \times C^w,\ t = 1,\ldots,n \tag{6}$$

$$D_t^s \le cf_t^{\,s} \times C^s,\ t = 1,\ldots,n \tag{7}$$

$$S_t^{es} \le C^{es},\ t = 1,\ldots,n \tag{8}$$

$$D_t^{es,\,discharge} \le S_t^{es} \times (1-\delta), 1,\ldots,n \tag{9}$$

$$S_{t+1}^{es} = (1-\delta) \times S_t^{es} + \eta \times D_t^{es,\,charge} - D_t^{es,\,discharge},\ t = 1\ to\ n\text{-}1 \tag{10}$$

$$S_1^{es} = (1-\delta) \times S_n^{es} + \eta \times D_n^{es,\,charge} - D_n^{es,\,discharge} \tag{11}$$

$$\sum_t D_t^{unmet} \le \delta \times \sum_t d_t^{\,unmet} \tag{12}$$

where superscripts *w*, *s*, and *es* denote wind, solar, and energy storage, respectively, and subscript *t* represents an hour in a year. The variables cf_t^w and cf_t^s are hourly capacity factors for wind and solar resources based on the MERRA-2 reanalysis data and d_t represents hourly electricity demand for CONUS. Constant δ is the loss rate for energy stored in energy storage, the constant η is the round-trip efficiency of energy storage, and the constant δ represents the maximum allowed fraction of electricity demand not met (for instance, a system with resource adequacy of at least 99.97% would have $\delta = 0.0003$). Constants, acc^w, acc^s, and acc^{es} represent the annualized capital cost for wind power, solar power, and energy storage, as defined previously. Constant m denotes the economic penalty for not meeting electricity demand. In this study, the economic penalty is set as $m = 0$.

The decision variables include hourly dispatched electricity from wind and solar generation assets at hour *t*, D_t^w and D_t^s; deployed capacity of wind and solar assets, C^w and C^s; discharged energy (from the grid to energy storage) and charged energy (from energy storage to the grid), $D_t^{es,discharge}$ and $D_t^{es,charge}$; deployed capacity of energy storage, C^{es}; and finally, electricity load not met by either variable renewable electricity (VRE) or discharged electricity from energy storage, D_t^{unmet}. Energy stored in energy storage, S_t^{es}, is a state variable and is determined by the optimization as well. All these variables take non-negative values but are otherwise unconstrained in obtaining the least-cost solution for the specified types of generation and storage assets to be deployed.

The total system cost (4) is minimized by varying the decision variables for generation and storage assets, subject to fundamental physical constraints in (5)–(12). For simplicity, near-zero variable costs were assumed for wind, solar, and energy storage, so the objective function only consisted of fixed cost terms effectively. Equation (5) represents the energy balance constraint for the renewable electricity system. Equations (6) and (7) constrain renewable energy generation based on historical capacity factors, which in turn depends on weather data and the assumed technology. Equations (8)-(11) characterize the discharged energy, charged energy, and stored energy in energy storage, respectively. We assumed a steady-state operation of energy storage (11). Finally, Equation (12) represents the resource adequacy requirement for the whole system.

Hourly electricity costs, shown in Figure 3, were estimated using the Lagrange dual variable of the hourly energy balance constraint for the VRE/storage system (5) (Boyd and Vandenberghe, 2004).

The variable renewable electricity system consists of wind turbines and solar photovoltaics (PV). The model assumed exogenously weighted-average generation potential for both wind and solar (i.e., hourly 80-meter wind and hourly downwelling surface solar radiation) at a 60 km resolution across CONUS (Shaner et al., 2018). The wind turbine's power curve is calculated as a cubic function of wind speed if the wind speed is between 3-15 m/s, and a cubic function of the rated 15 m/s speed if the wind speed is between 15-25 m/s (Shaner et al., 2018). The capacity factors for wind and solar resources averaged over CONUS for the 36 years considered are 0.38 and 0.22, respectively. We note that the assumed weighted-average generation potential of wind and solar might lead to lower capacity factors than those reported in the recent wind and solar energy projects as we consider a much higher capacity of wind and solar energy which would inevitably be deployed in places with worse resources than those developed already. However, the need for energy storage is driven by the temporal and spatial correlation and variability of wind and solar generation as well as those of electricity demand, so the conclusions of our study are robust.

Actual hourly electricity load data for the United States between July 2015 and July 2016 were taken from (Shaner et al., 2018), which in turn were based on data from (U.S. Energy Information Administration (EIA), 2017). Existing power plants and emerging low-carbon electricity generation technologies, such as fossil fuel power plants with carbon capture and storage (CCS), bioenergy with carbon capture and storage (BECCS), geothermal, hydropower, and tidal power, are not considered in this study.

Table S1 presents the technology assumptions used herein. In the base case, the capital costs of wind turbines and solar PVs were both assumed to be $1,500/kW to highlight the geophysical characteristics of wind and solar resources

(National Renewable Energy Laboratory (NREL), 2018; U.S. Energy Information Administration (EIA), 2018a). A generic energy storage technology was assumed with a charging time of one hour (i.e., the energy constraint and power constraint were the same), a 90% round-trip efficiency, and a 1% loss in the stored energy per a year of storage (Zerrahn and Schill, 2017).

Algorithm for Analyzing Storage Utilization

We showed the utilization pattern of energy storage in Figure 6 in the main text. The algorithm used for performing this analysis is listed in **Box S1**.

First, we ran the optimization as described above. We then took four decision variables from the optimization results: (1) the optimized deployed storage capacity, C^{es}; (2) the optimized discharged energy from storage (from the grid to energy storage), $D_t^{es,discharge}$; (3) the optimized charged energy storage (from energy storage to the grid), $D_t^{es,charge}$; and (4) the energy stored in storage at 0th time step, S_0^{es}.

We then performed a parametric analysis on storage capacity to quantify what fractions of optimal discharged energy could be met if only a fraction of the optimal storage capacity were available. For a given fraction of optimal storage capacity (α), the algorithm involves the following steps decribed in **Box S1**. To distinguish variables, we denoted variables associated with the resized storage capacity with ~.

(1) At t = 0, set the energy stored in storage as $\tilde{S}_0^{es} = S_0^{es}$.

(2) For t = 1 to N-1, where N is the total number of hours in a year:

Determine if charge or discharge happens for the optimal storage at time t.

If a charge happens, the energy stored in the resized storage is calculated as

$$\tilde{S}_t^{es} = \min\left\{ \tilde{S}_{t-1}^{es} + \eta \times D_t^{es,charge}, \alpha \times C^{es} \right\}$$

and the charged energy is calculated as $\tilde{S}_t^{es} - \tilde{S}_{t-1}^{es}$

If a discharge happens, the energy stored in the resized storage is calculated as

$$\tilde{S}_t^{es} = \max\left\{ \tilde{S}_{t-1}^{es} - D_t^{es,discharge}, 0 \right\}$$

and the discharged energy is calculated as $\tilde{S}_{t-1}^{es} - \tilde{S}_t^{es}$

(3) When (2) is finished, we can calculate the total discharged energy for the resized storage as

$\tilde{d}^{es} = \sum_{t=1}^{N} \left(\tilde{S}_{t-1}^{es} - \tilde{S}_t^{es} \right)$. By definition, $0 \le \tilde{d}^{es} \le d^{es}$, where d^{es} is the total discharged energy for the optimal storage.

Box S1. Algorithm for analyzing storage utilization. Related to Figure 6.

Additional Results for Technology Scenarios

We modeled five additional technology scenarios (**Table S2**) to explore the impact of VRE costs and availability of generation technologies on our findings. For these technology scenarios, we assumed the actual electricity demands for all hours must be met (i.e., 100% resource adequacy requirement). The optimization results for these technology scenarios are shown in **Figures S1–S18**. A selection of numerical results for the least-cost system for 2015 is shown in **Table S3**.

Additional Results for Resource Adequacy Scenarios

In the previous results, the variable renewable electricity and storage (VRE/storage) system was optimized for a 100% resource adequacy. In other words, electricity demand for all hours is met by the VRE/storage system. In reality, the actual electricity system strives to high reliability but cannot eliminate power outage. For instance, the resource adequacy requirement of power systems in the United States is required to be at least 99.97% (Federal Energy Regulatory Commission, 2010). Here, we investigated the impact of resource adequacy of at least 99.97% (rather than 100%). In this case, we changed the value of δ from 0 to 0.0003 but kept all the other settings in the optimization (Equation (4)-(12)). The optimization results for the 99.97% resource adequacy are shown in **Figures S19–S29**. To facilitate a comparison of results at these two resource adequacy criteria (100% vs. 99.7%) for the year 2015, we summarized results in **Table S4** and reported the relative difference in the optimized results for these two resource adequacy cases in **Table S5**.

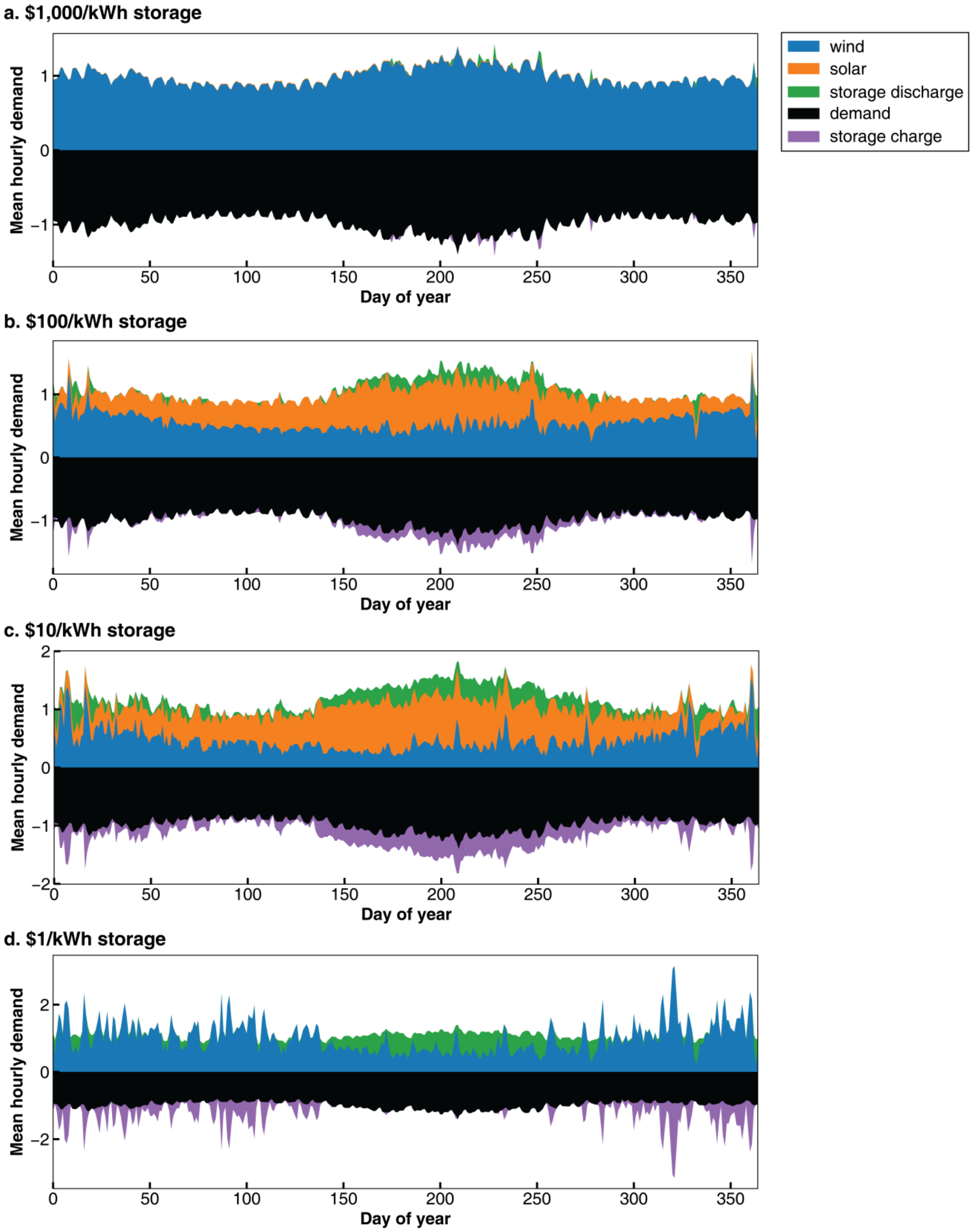

Figure S1. Daily-average least-cost dispatch mix for the baseline VRE/storage system for the year 2015 at different storage costs: $1000/kWh (a), $100/kWh (b), $10/kWh (c), and $1/kWh (d). The least-cost dispatch mix at $1/kWh and that at $0.1/kWh storage costs are the same. **Related to Figure 1, Figure 2, and Figure 3.**

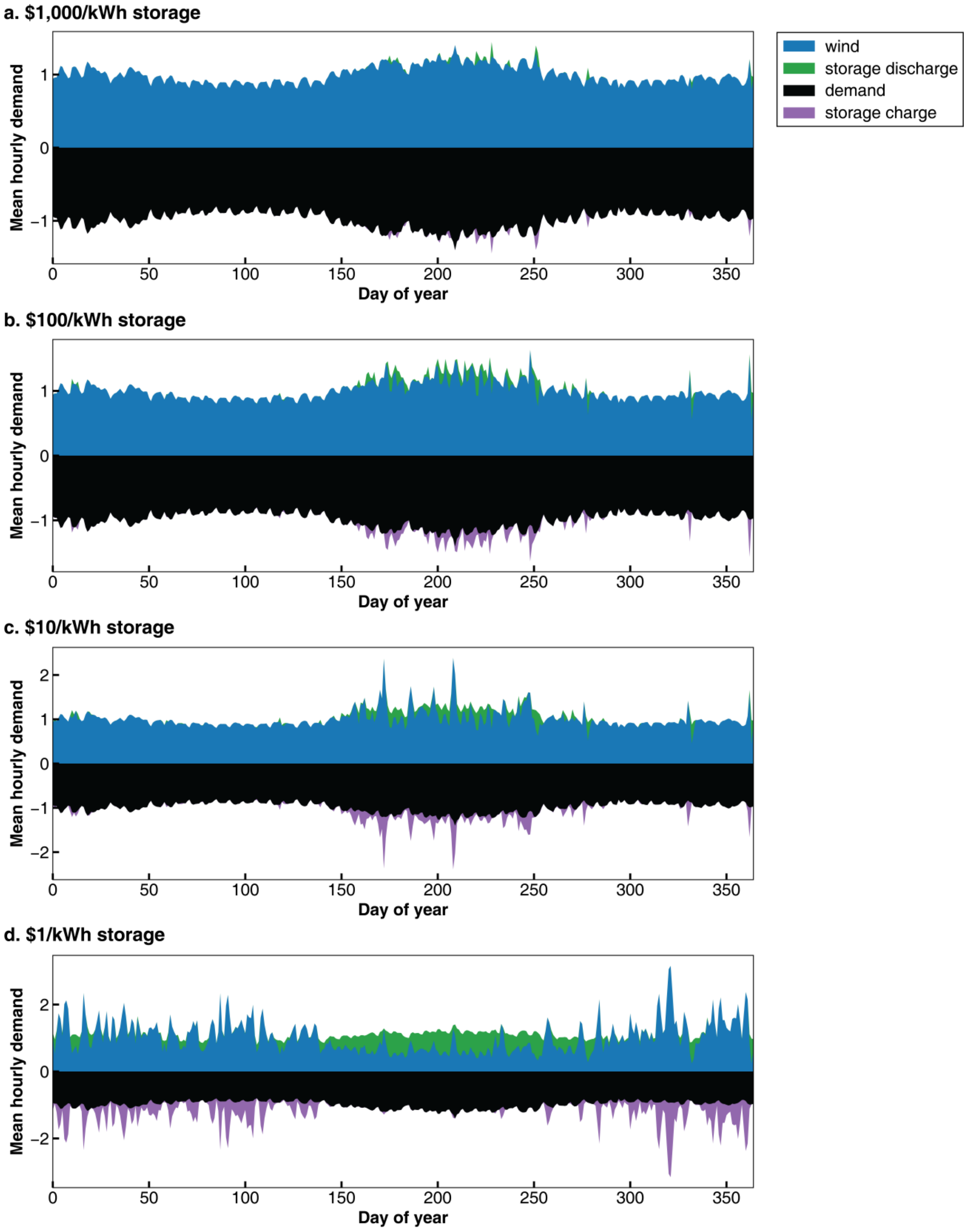

Figure S2. Daily-average least-cost dispatch mix for the wind/storage system for the year 2015 at different storage costs: $1000/kWh (a), $100/kWh (b), $10/kWh (c), and $1/kWh (d). The least-cost dispatch mix at $1/kWh and that at $0.1/kWh storage costs are the same. **Related to Figure 3 and Figure S1.**

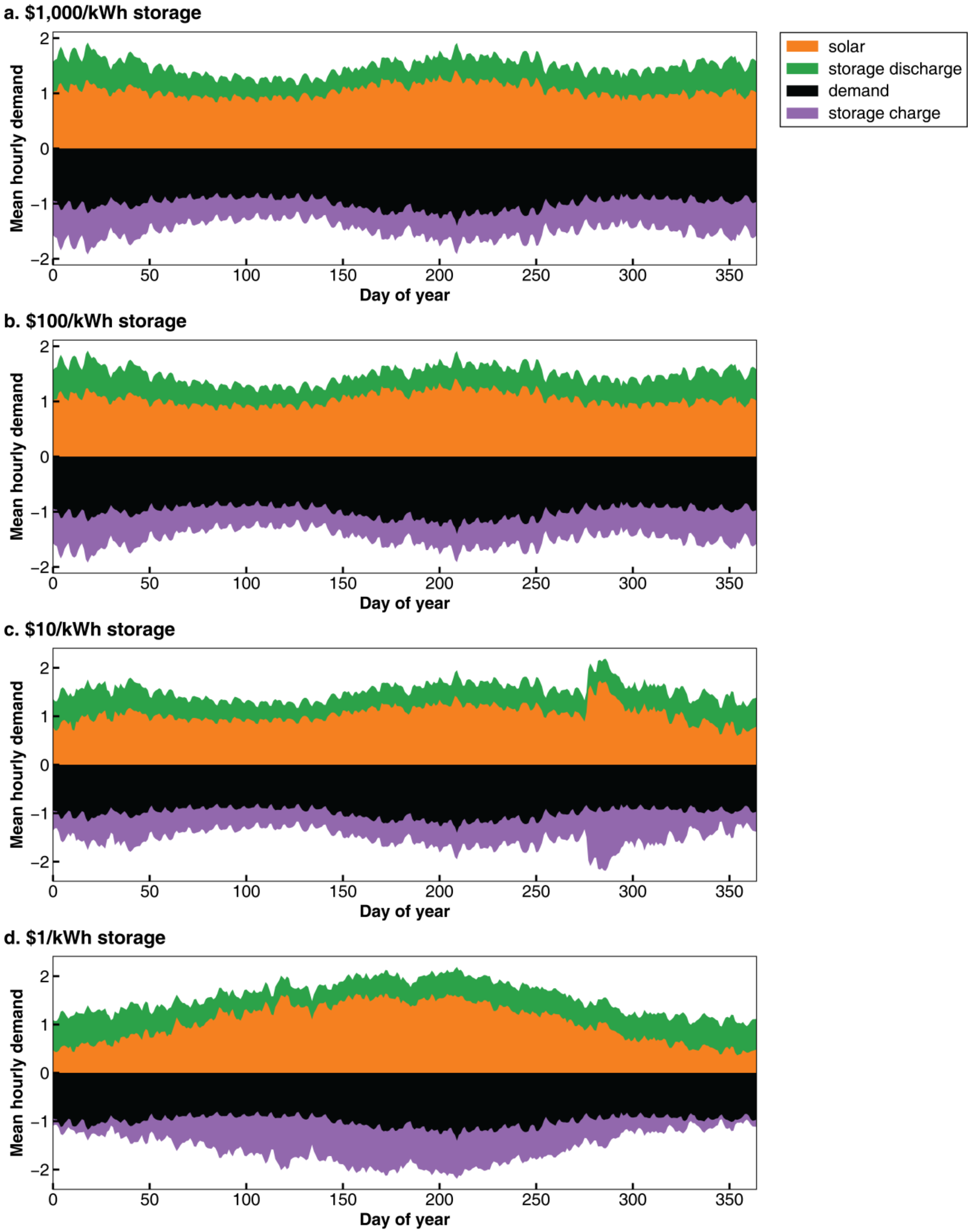

Figure S3. Daily-average least-cost dispatch mix for the solar/storage system for the year 2015 at different storage costs: $1000/kWh (a), $100/kWh (b), $10/kWh (c), and $1/kWh (d). The least-cost dispatch mix at $1/kWh and that at $0.1/kWh storage costs are the same. **Related to Figure 3 and Figure S1.**

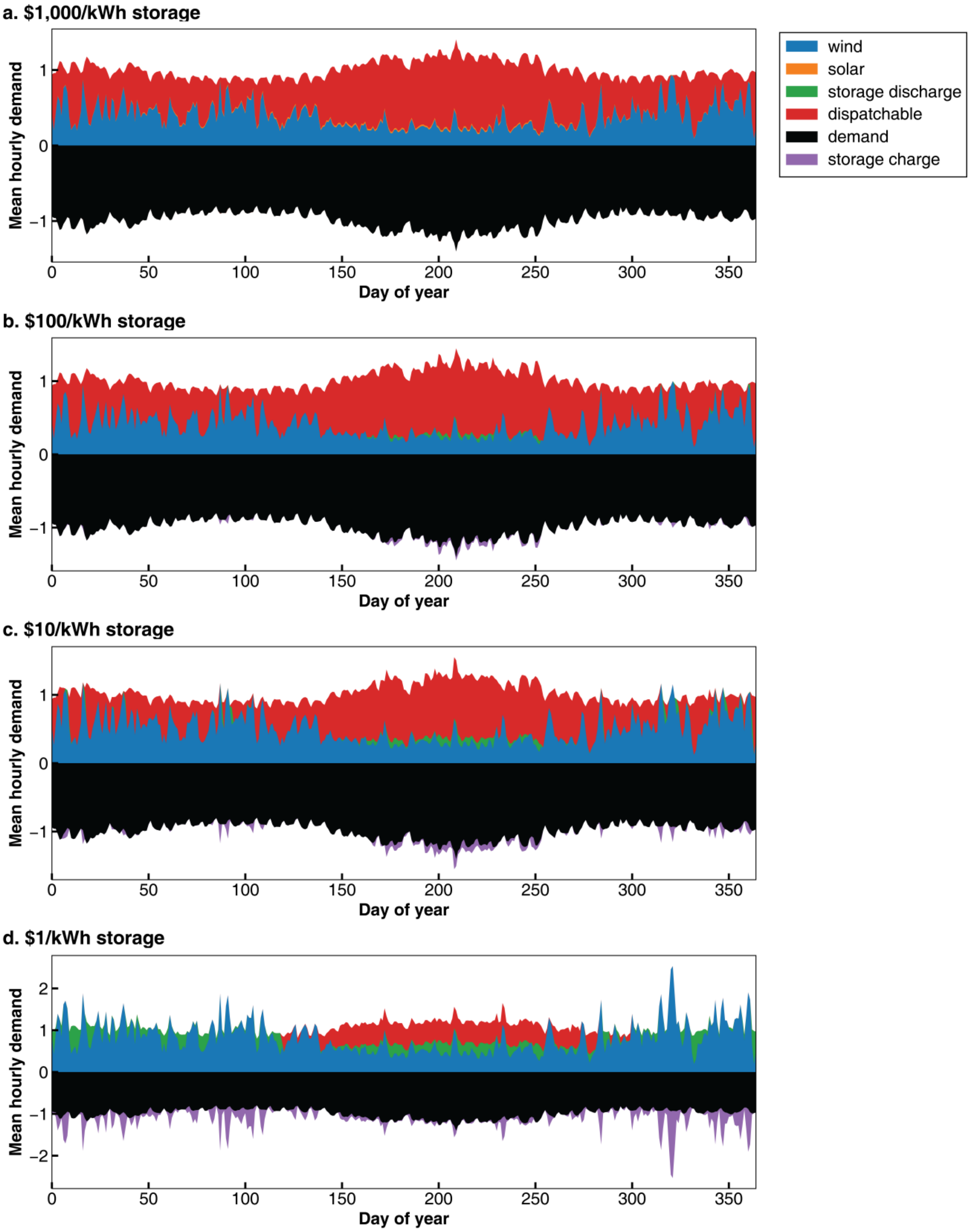

Figure S4. Daily-average least-cost dispatch mix for the wind/solar/storage/dispatchable generation system for the year 2015 at different storage costs: \$1000/kWh (a), \$100/kWh (b), \$10/kWh (c), and \$1/kWh (d). The least-cost dispatch mix at \$1/kWh and that at \$0.1/kWh storage costs are similar but not exactly the same. **Related to Figure S1 and Figure S11.**

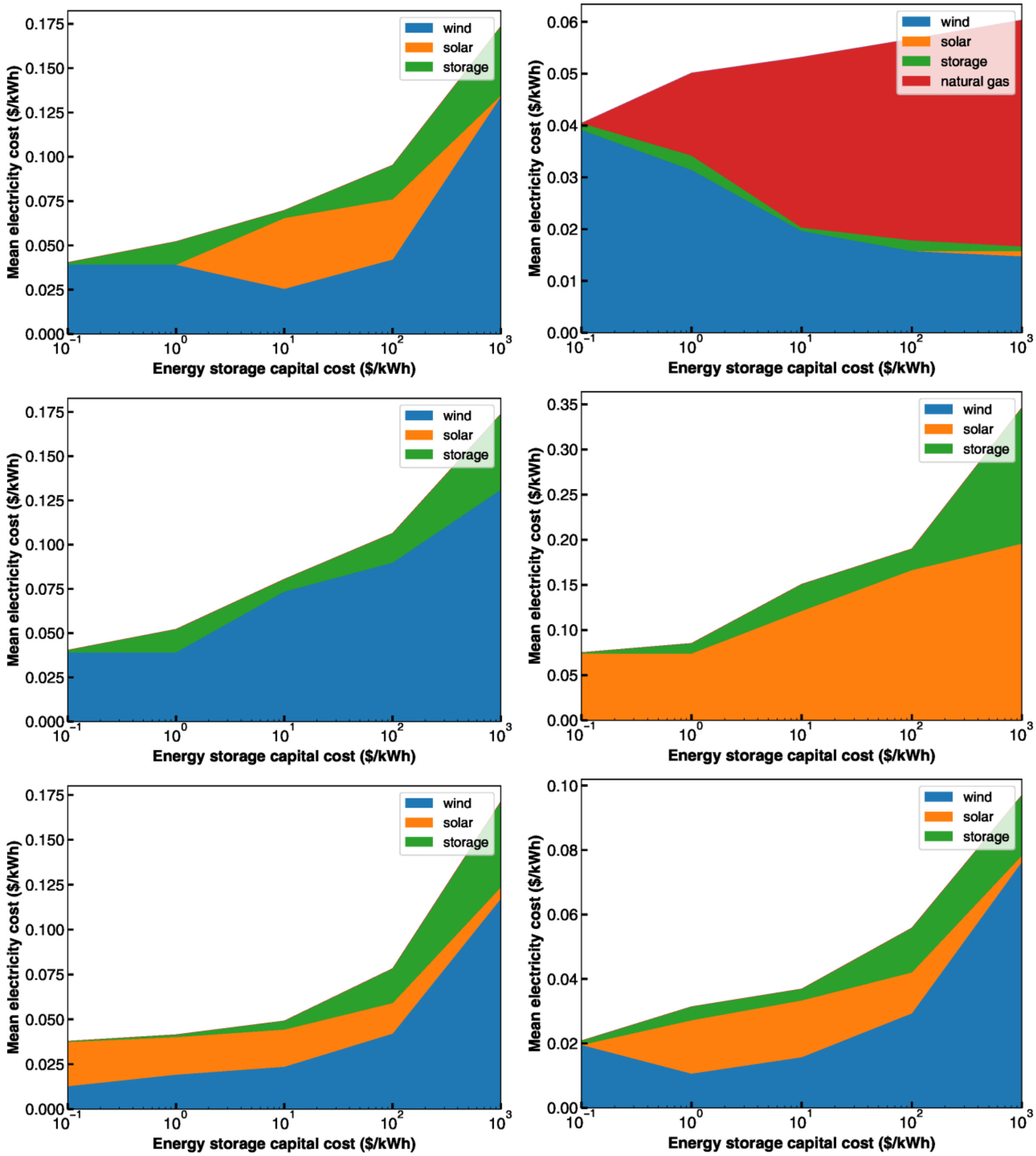

Figure S5. Effect of storage cost on the mean electricity cost of the least-cost VRE/storage systems for the year 2015. Each panel shows optimization results for a technology scenario in Table S2: “baseline” (top left), “baseline + dispatchable generation (natural gas)” (top right), “wind only” (middle left), “solar only” (middle right), “wind and cheaper solar” (bottom left), and “cheaper wind and cheaper solar” (bottom right). **Related to Figure 1.**

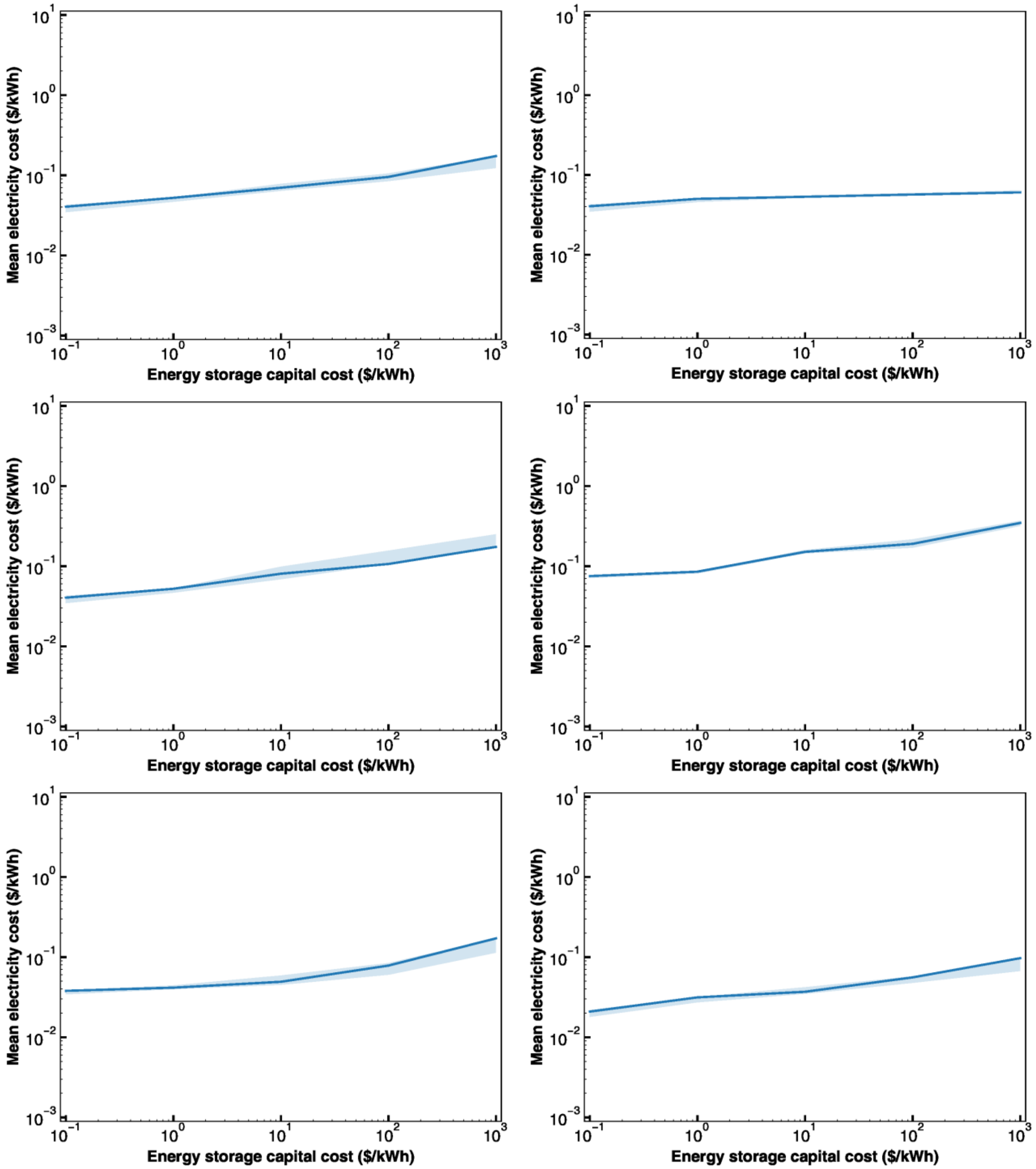

Figure S6. Effect of storage cost on the mean electricity cost of the least-cost VRE/storage systems. Bold lines show optimization results for the year 2015, and shaded areas show the range of results from independent annual optimizations (1980–2015). Each panel shows optimization results for a technology scenario in Table S2: “baseline” (top left), “baseline + dispatchable generation (natural gas)” (top right), “wind only” (middle left), “solar only” (middle right), “wind and cheaper solar” (bottom left), and “cheaper wind and cheaper solar” (bottom right). **Related to Figure 1.**

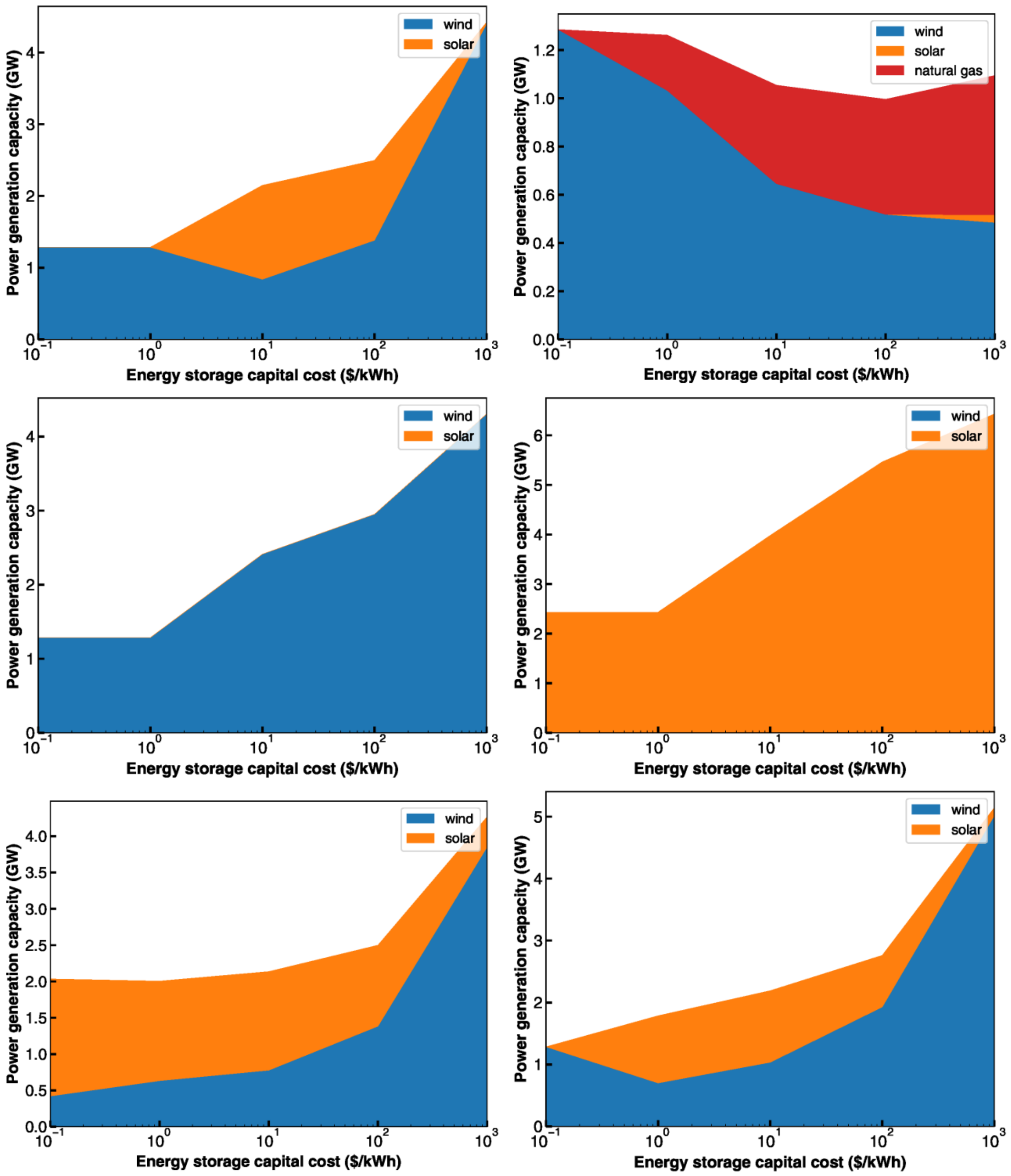

Figure S7. Effect of storage cost on the deployed wind and solar capacity in the least-cost VRE/storage systems for the year 2015. Results for the deployed storage capacity are in Figure S13. Each panel shows optimization results for a technology scenario in Table S2: "baseline" (top left), "baseline + dispatchable generation (natural gas)" (top right), "wind only" (middle left), "solar only" (middle right), "wind and cheaper solar" (bottom left), and "cheaper wind and cheaper solar" (bottom right). **Related to Figure 2.**

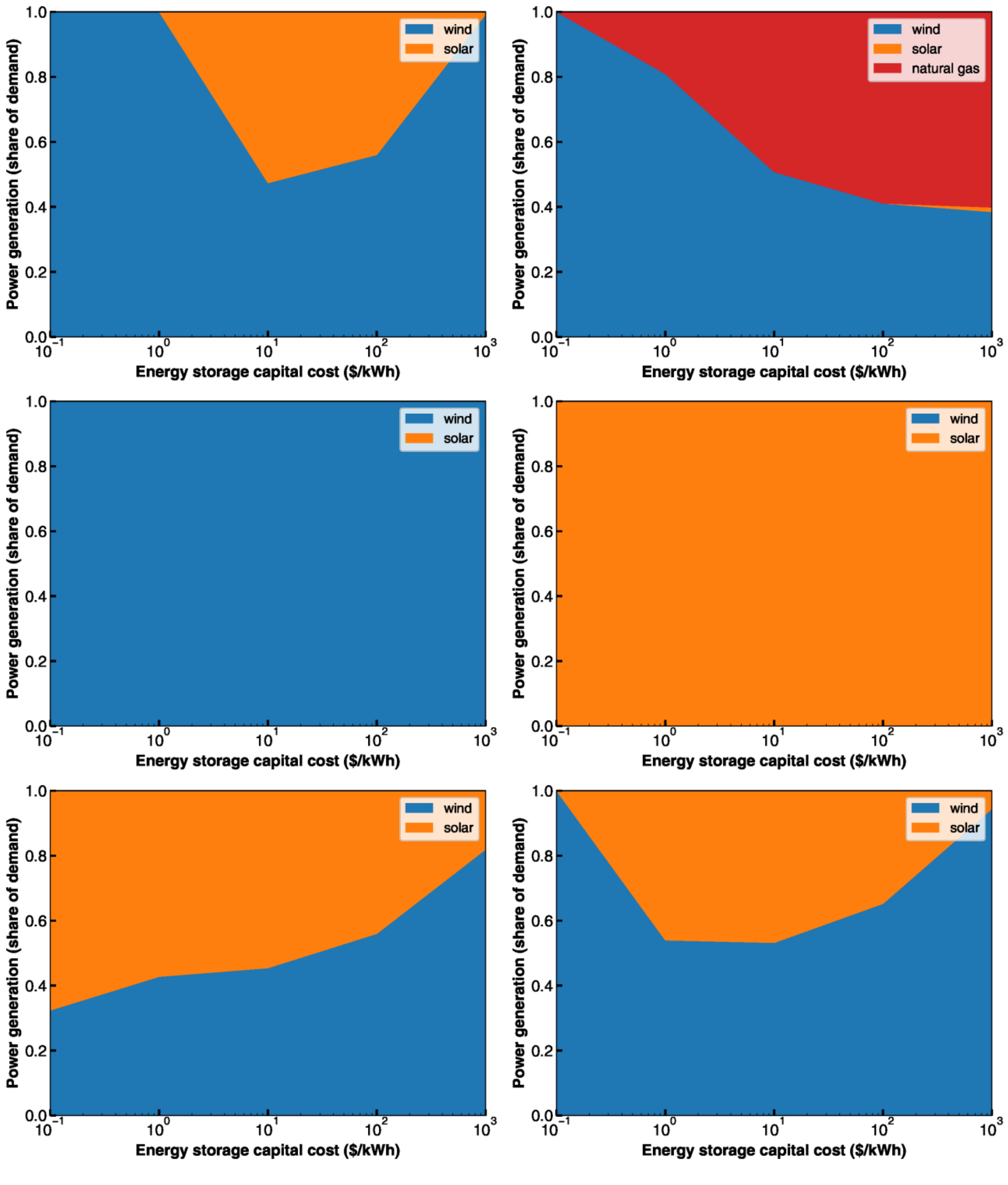

Figure S8. Effect of storage cost on relative shares of net electricity generation in the least-cost VRE/storage systems for the year 2015. Curtailed generation of wind and solar electricity is excluded in this figure but available in Figure S10. Each panel shows optimization results for a technology scenario in Table S2: "baseline" (top left), "baseline + dispatchable generation (natural gas)" (top right), "wind only" (middle left), "solar only" (middle right), "wind and cheaper solar" (bottom left), and "cheaper wind and cheaper solar" (bottom right). **Related to Figure 2.**

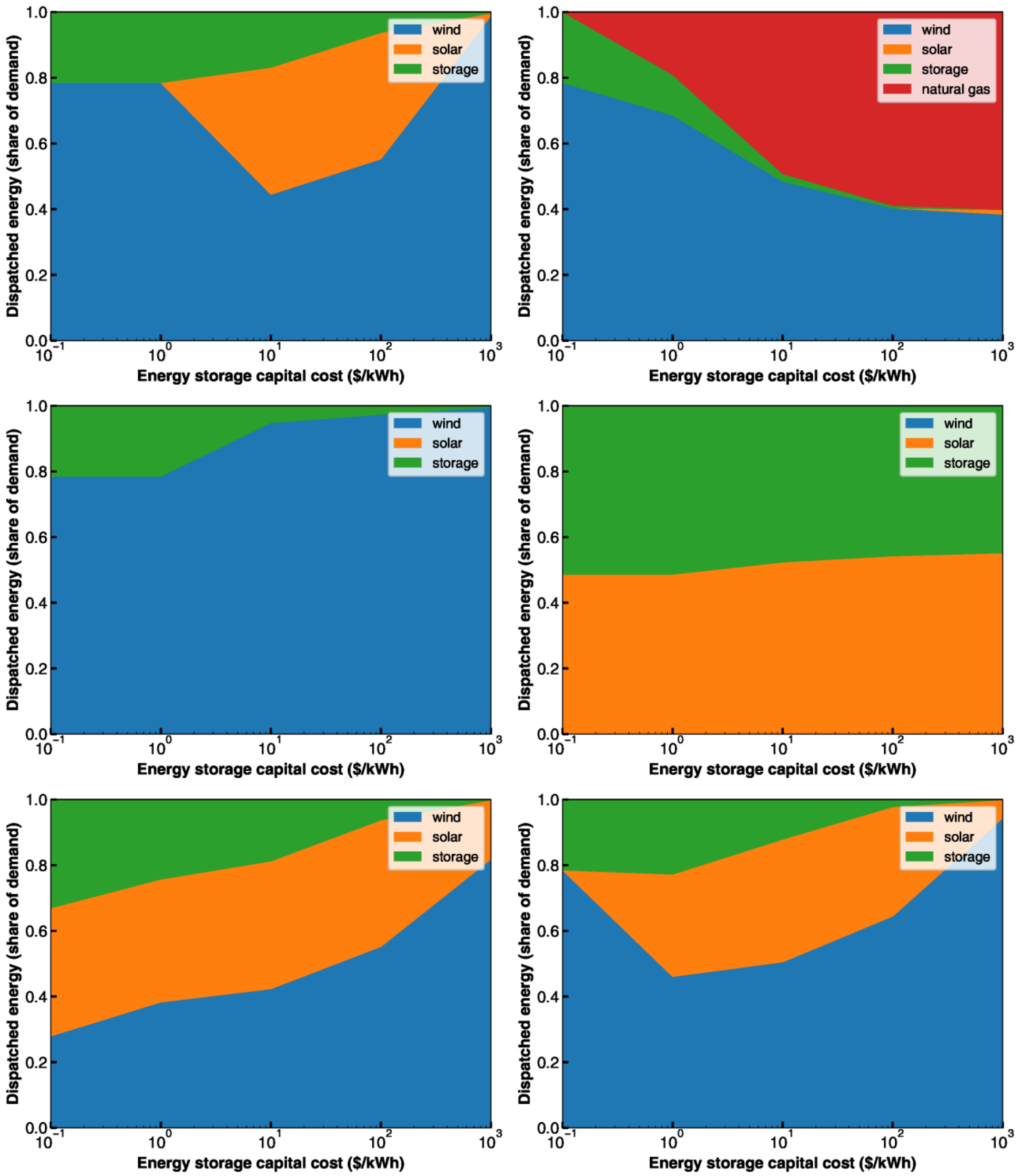

Figure S9. Effect of storage cost on relative shares of dispatched electricity (that directly meets the electricity demand) in the least-cost VRE/storage systems for the year 2015. Curtailed generation of wind and solar electricity is excluded in this figure but available in Figure S10. Each panel shows optimization results for a technology scenario in Table S2: "baseline" (top left), "baseline + dispatchable generation (natural gas)" (top right), "wind only" (middle left), "solar only" (middle right), "wind and cheaper solar" (bottom left), and "cheaper wind and cheaper solar" (bottom right). **Related to Figure 2, and Figure S1-S4.**

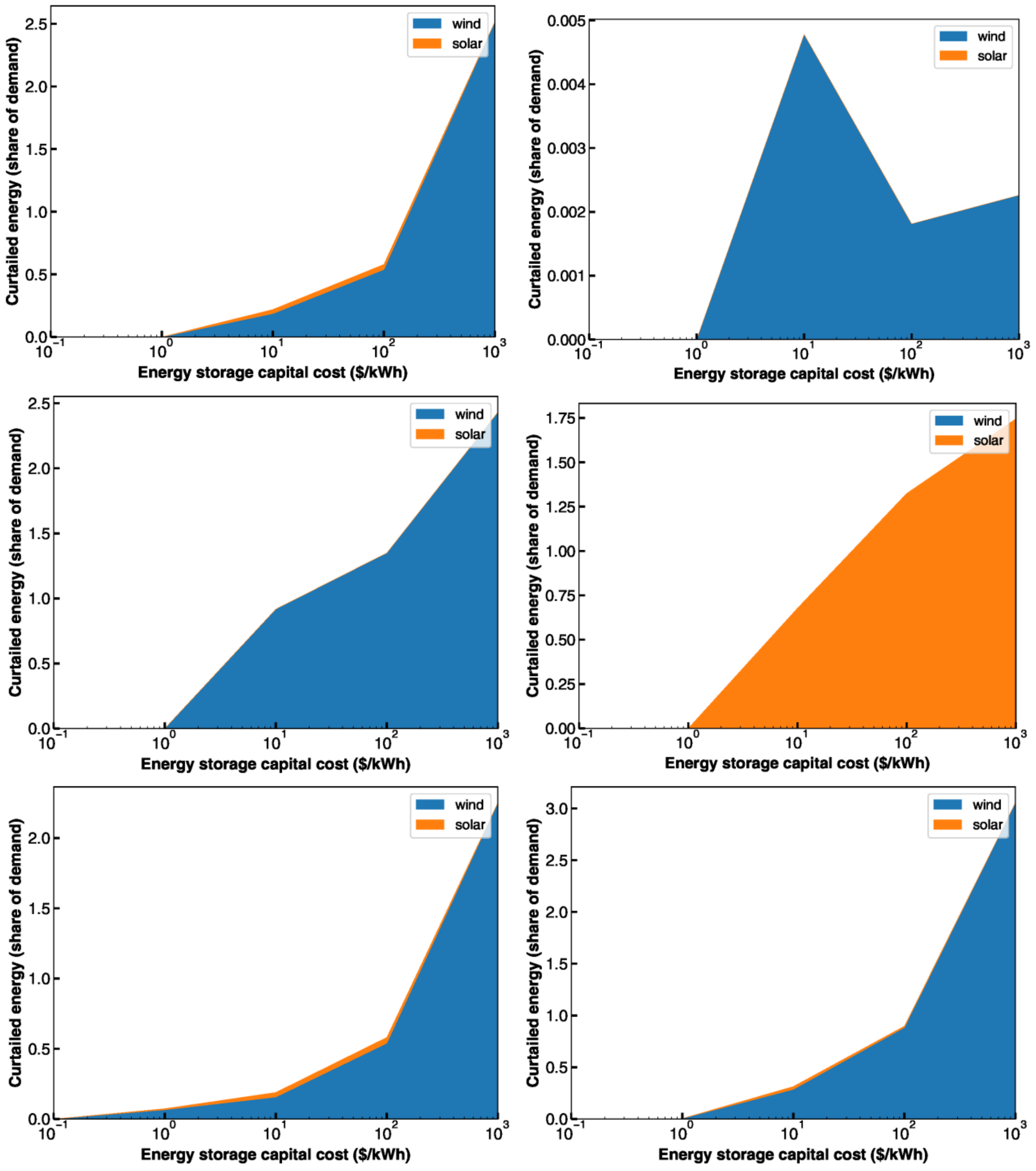

Figure S10. Effect of storage cost on the curtailed generation of wind and solar electricity in the least-cost VRE/storage systems for the year 2015. Each panel shows optimization results for a technology scenario in Table S2: “baseline” (top left), “baseline + dispatchable generation (natural gas)” (top right), “wind only” (middle left), “solar only” (middle right), “wind and cheaper solar” (bottom left), and “cheaper wind and cheaper solar” (bottom right). **Related to Figure 2.**

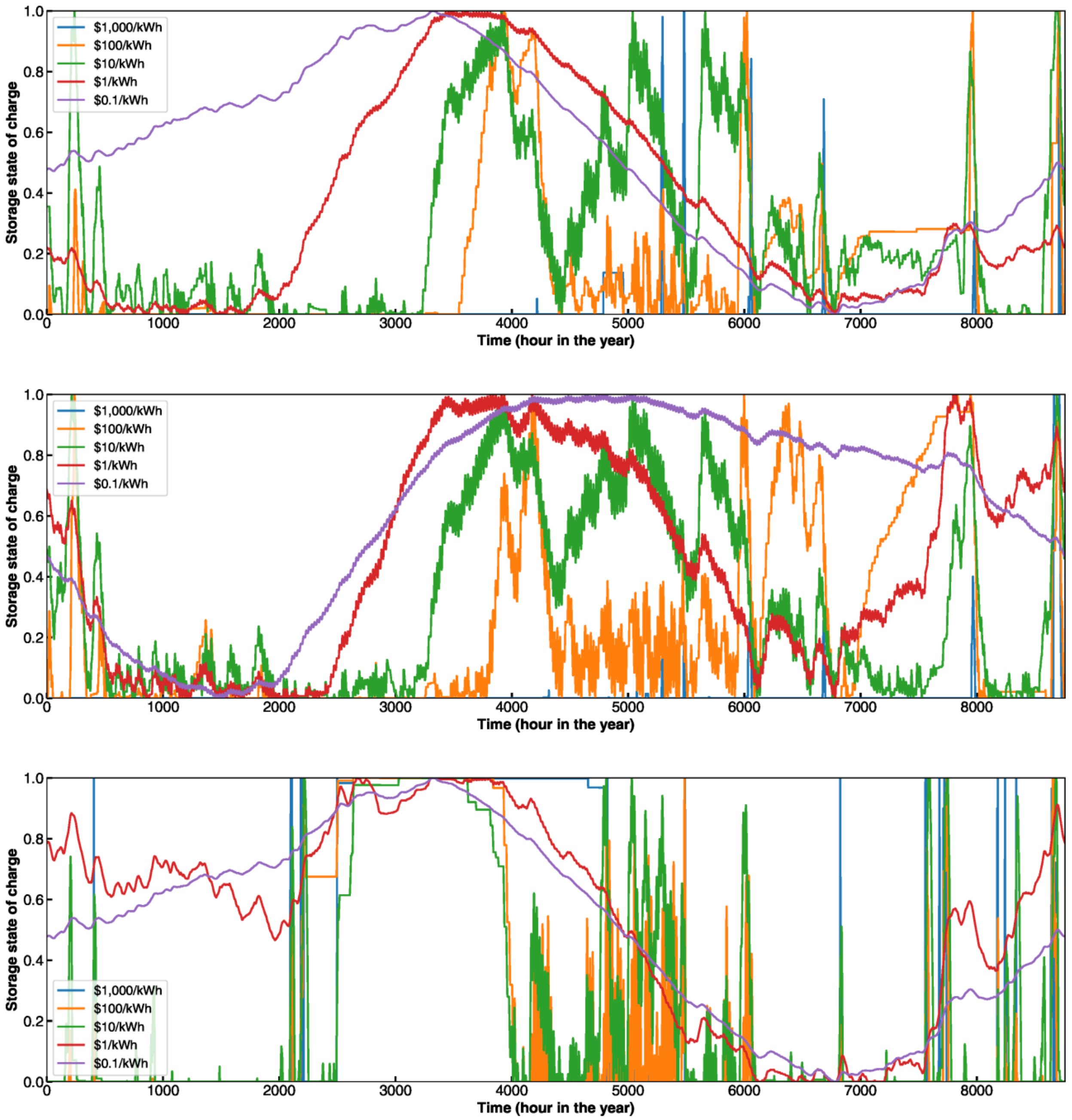

Figure S11. Effect of storage cost on the state-of-charge for energy storage in the least-cost VRE/storage systems for the year 2015. From top to bottom, each panel shows optimization results for a technology scenario in Table S2: "wind and cheaper solar" (top), and "cheaper wind and cheaper solar" (middle), and "baseline + dispatchable generation (natural gas)" (bottom). **Related to Figure 3 and Figure S4.**

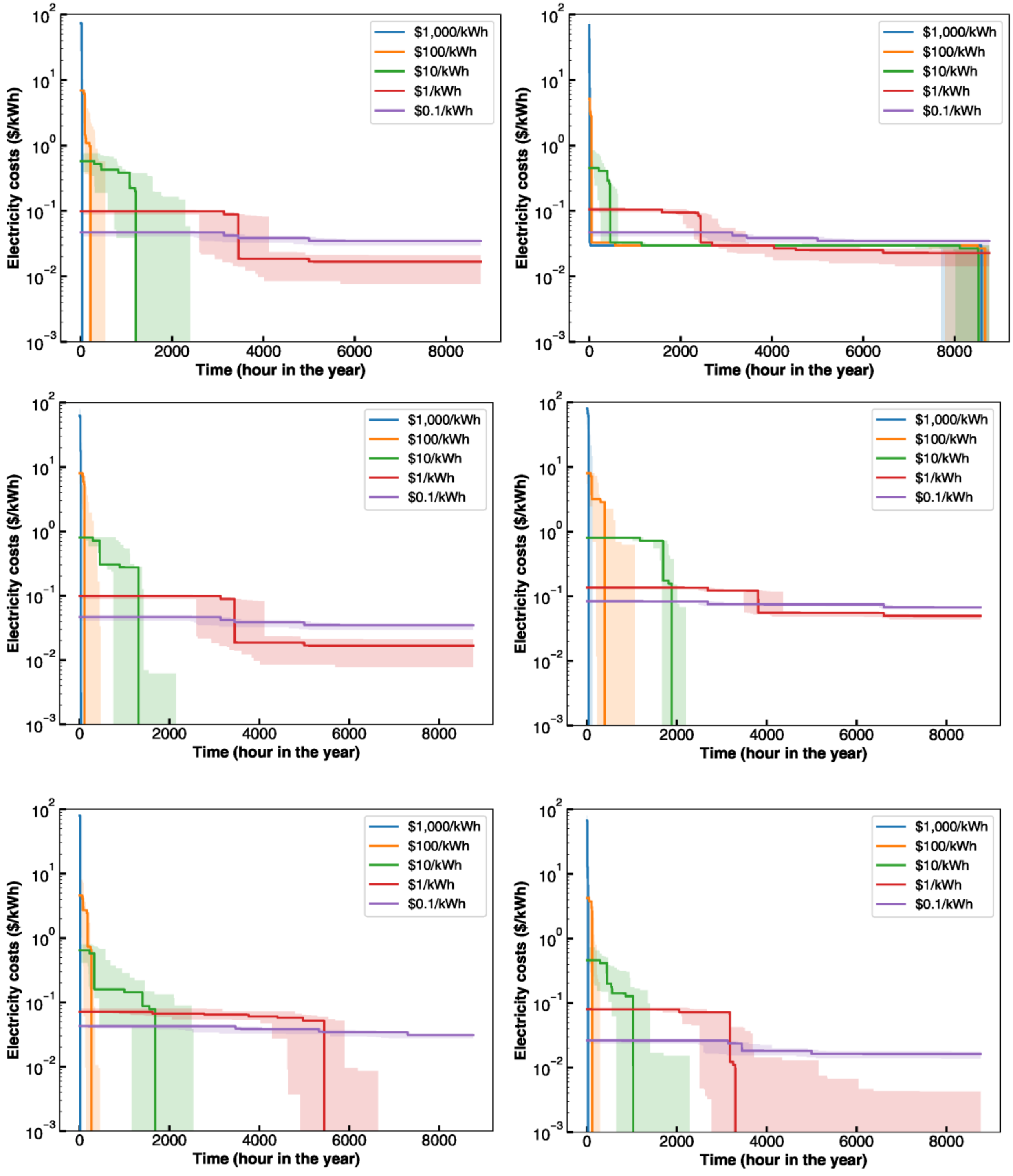

Figure S12. Effect of storage cost on the cost duration curve for the least-cost VRE/storage systems. Bold lines show optimization results for the year 2015, and shaded areas show the range of results from independent annual optimizations (1980–2015). Each panel shows optimization results for a technology scenario in Table S2: “baseline” (top left), “baseline + dispatchable generation (natural gas)” (top right), “wind only” (middle left), “solar only” (middle right), “wind and cheaper solar” (bottom left), and “cheaper wind and cheaper solar” (bottom right). **Related to Figure 4.**

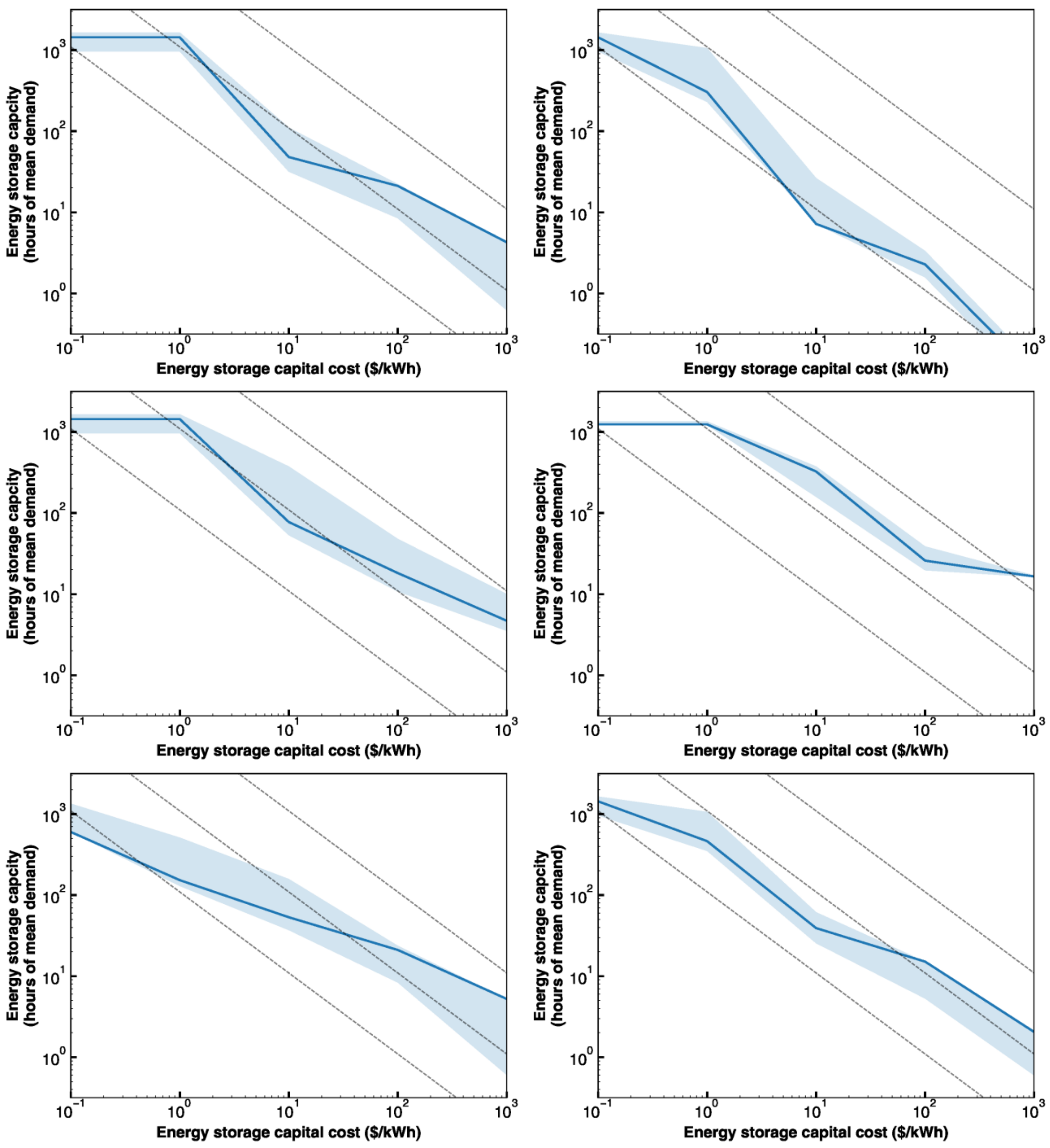

Figure S13. Effect of storage cost on the deployed storage capacity in the least-cost VRE/storage systems. Bold lines show optimization results for the year 2015, and shaded areas show the range of results from independent annual optimizations (1980–2015). Each panel shows optimization results for a technology scenario in Table S2: "baseline" (top left), "baseline + dispatchable generation (natural gas)" (top right), "wind only" (middle left), "solar only" (middle right), "wind and cheaper solar" (bottom left), and "cheaper wind and cheaper solar" (bottom right). Dashed lines are the same as those shown in Figure 5a. For reference, the hourly mean electricity demand for the US is 449,423 MWh in the year 2015. **Related to Figure 5.**

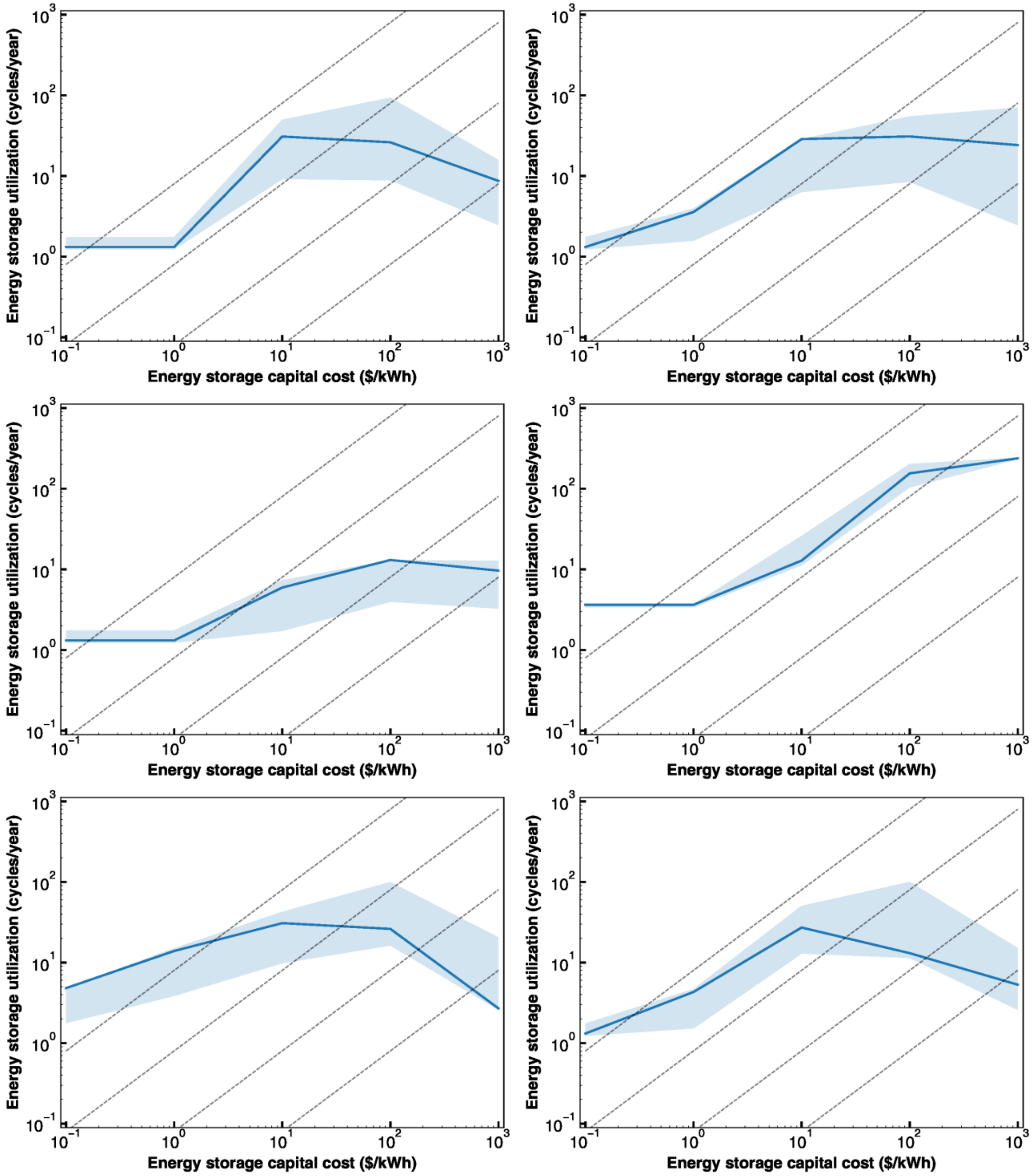

Figure S14. Effect of storage cost on the utilization of storage in the least-cost VRE/storage systems. Bold lines show optimization results for the year 2015, and shaded areas show the range of results from independent annual optimizations (1980–2015). Each panel shows optimization results for a technology scenario in Table S2: "baseline" (top left), "baseline + dispatchable generation (natural gas)" (top right), "wind only" (middle left), "solar only" (middle right), "wind and cheaper solar" (bottom left), and "cheaper wind and cheaper solar" (bottom right). Dashed lines are the same as those shown in Figure 5b. **Related to Figure 5.**

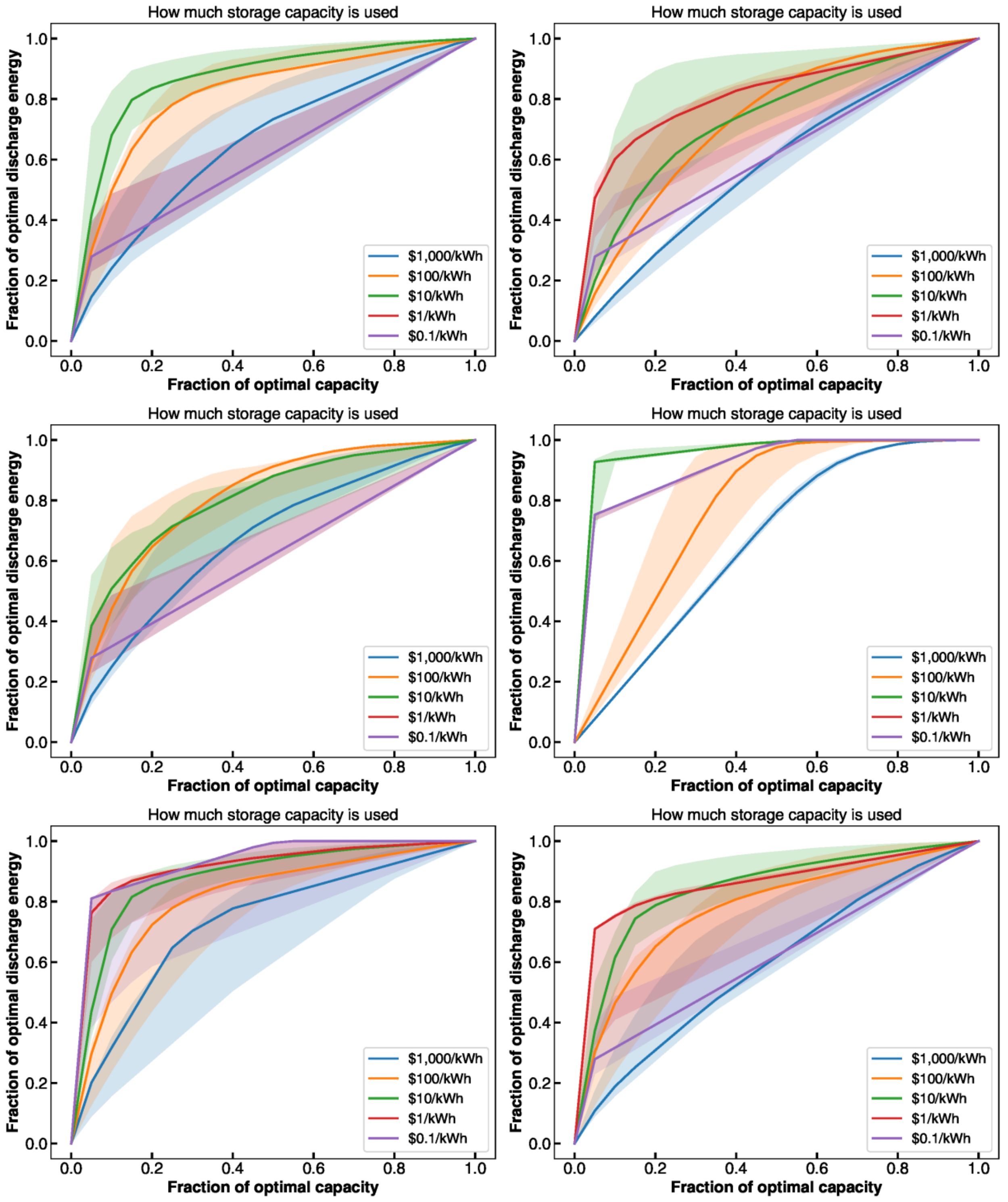

Figure S15. Relationships of storage discharged energy and storage capacity at different storage costs. Bold lines show optimization results for the year 2015, and shaded areas show the range of results from independent annual optimizations (1980–2015). Each panel shows optimization results for a technology scenario in Table S2: "baseline" (top left), "baseline + dispatchable generation (natural gas)" (top right), "wind only" (middle left), "solar only" (middle right), "wind and cheaper solar" (bottom left), and "cheaper wind and cheaper solar" (bottom right). **Related to Figure 6.**

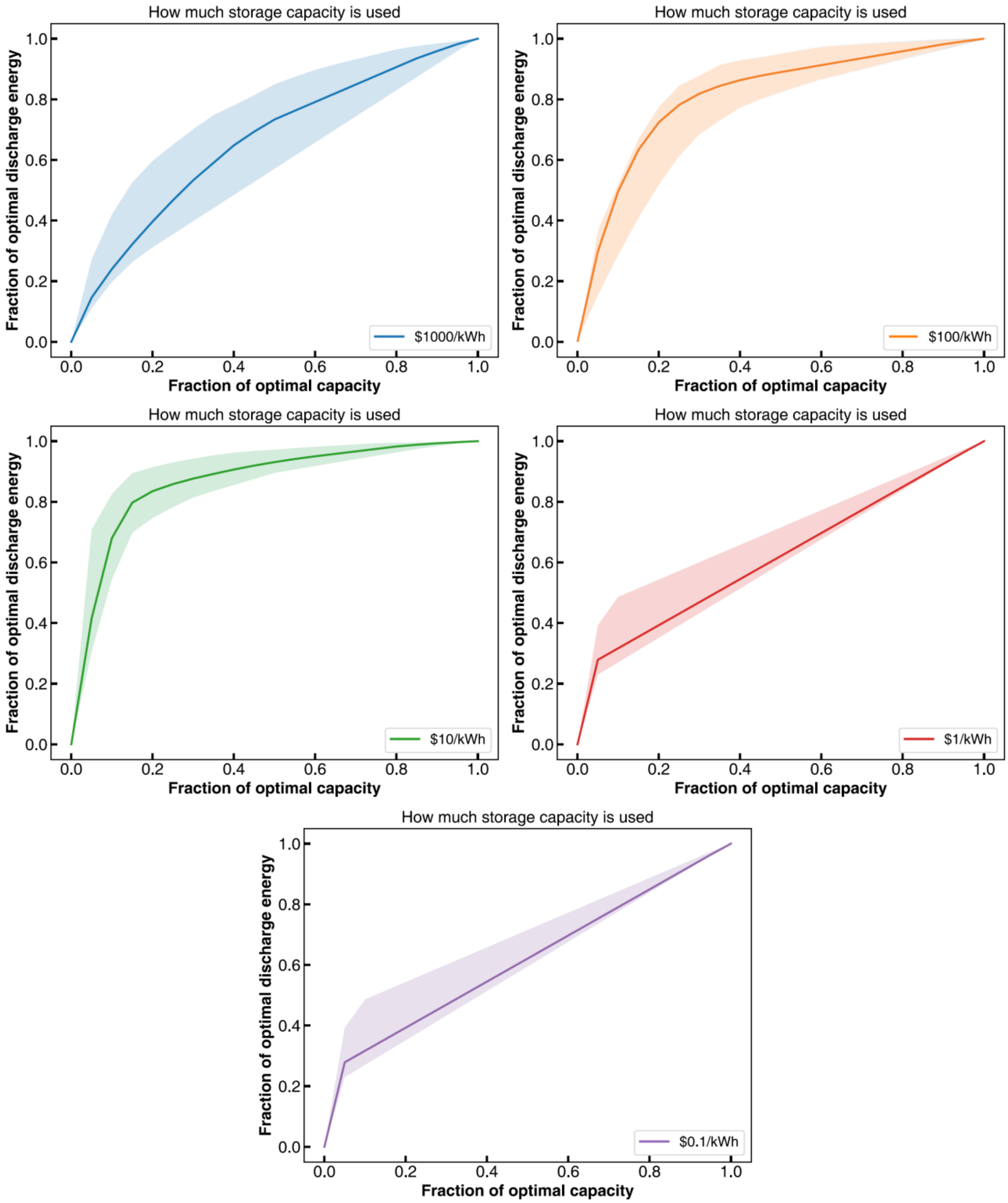

Figure S16. Relationships of discharged energy from storage and storage capacity for the baseline VRE/storage system at different storage costs. Bold lines show optimization results for the year 2015, and shaded areas show the ranges of results of independent optimizations for each of 36 years (1980–2015). Each panel shows optimal results for the VRE/storage system at an assumed storage cost. **Related to Figure 6.**

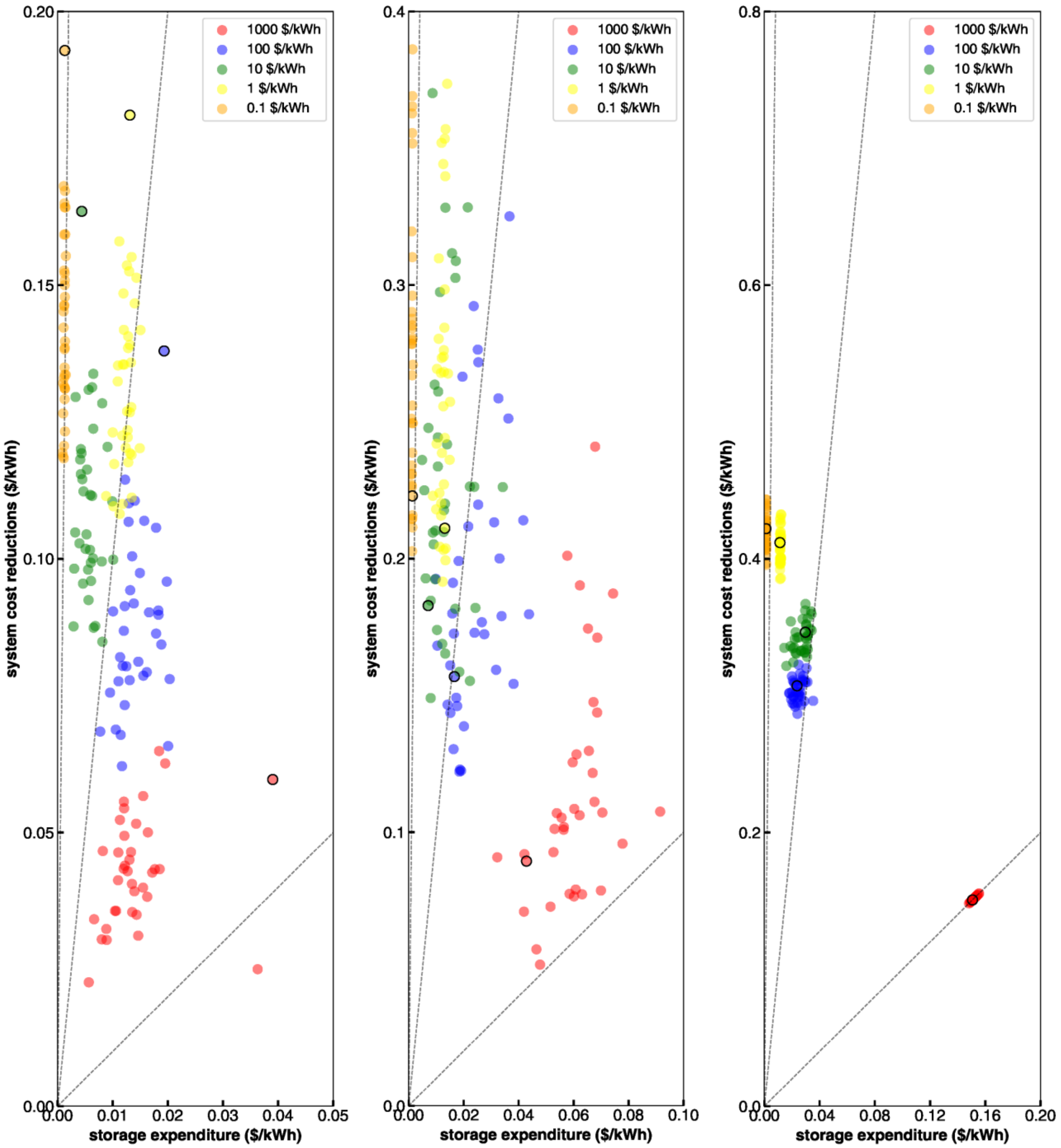

Figure S17. Relationships of system cost reductions and energy storage expenditures for the least-cost VRE/storage systems at different storage costs. Each point represents the results of an independent annual optimization (1980–2015) at an assumed energy storage cost. Points with bold black borders indicate the results for the year 2015. The panels show results for three technology scenarios: “baseline” (left), “wind only” (middle), and “solar only” (right). For the “solar only” scenario, the requirement of 100% resource adequacy cannot be met without the use of energy storage since there is no solar electricity generation at night every day. We use a case with a $2,000/kWh energy storage as the baseline to calculate system cost reductions. We use different x-axis and y-axis scales across figures. **Related to Figure 7.**

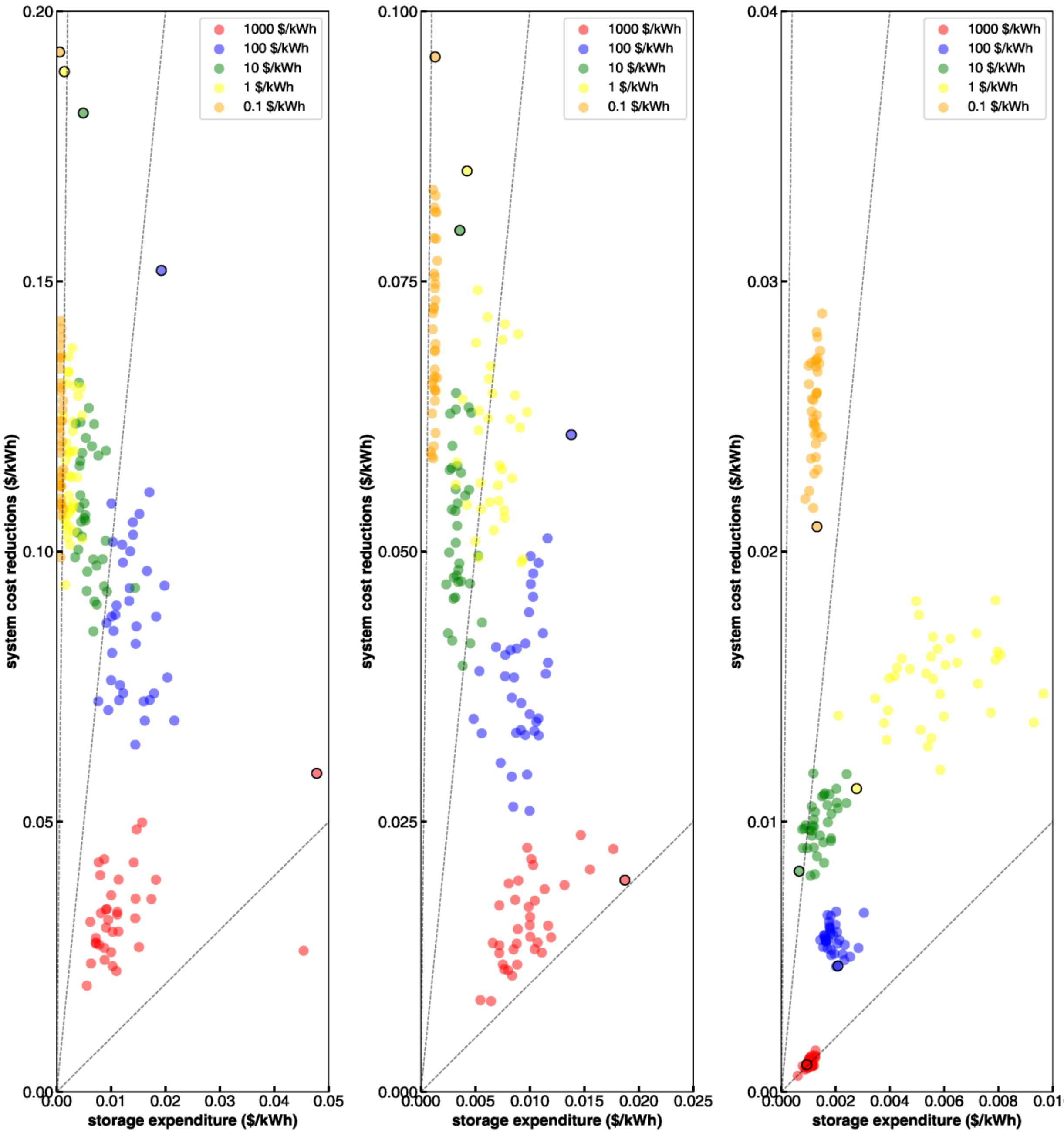

Figure S18. Relationships of system cost reductions and energy storage expenditures for the least-cost VRE/storage systems at different storage costs. Each point represents the results of an independent annual optimization (1980–2015) at an assumed energy storage cost. Points with bold black borders indicate the results for the year 2015. The panels show results for three technology scenarios: “wind and cheaper solar” (left), “cheaper wind and cheaper solar” (middle), and “baseline + dispatchable generation (natural gas)” (right). We use different x-axis and y-axis scales across figures. **Related to Figure 7.**

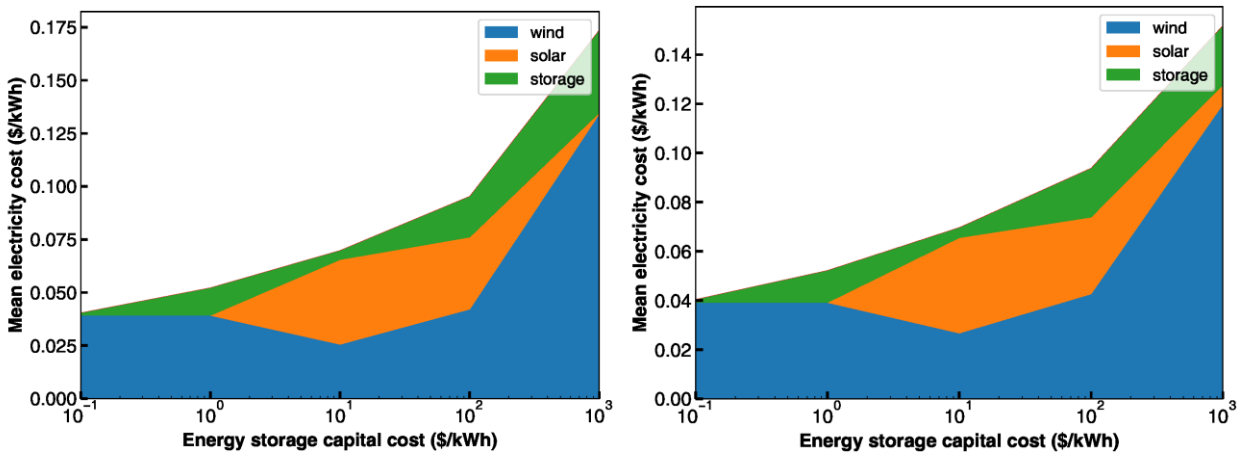

Figure S19. Effect of storage cost on the mean electricity cost of the least-cost VRE/storage systems for the year 2015. The baseline technology scenario (Table S2) is assumed. The left panel shows the optimization results for 100% resource adequacy, and the right panel shows those for at least 99.97% resource adequacy. **Related to Figure 1.**

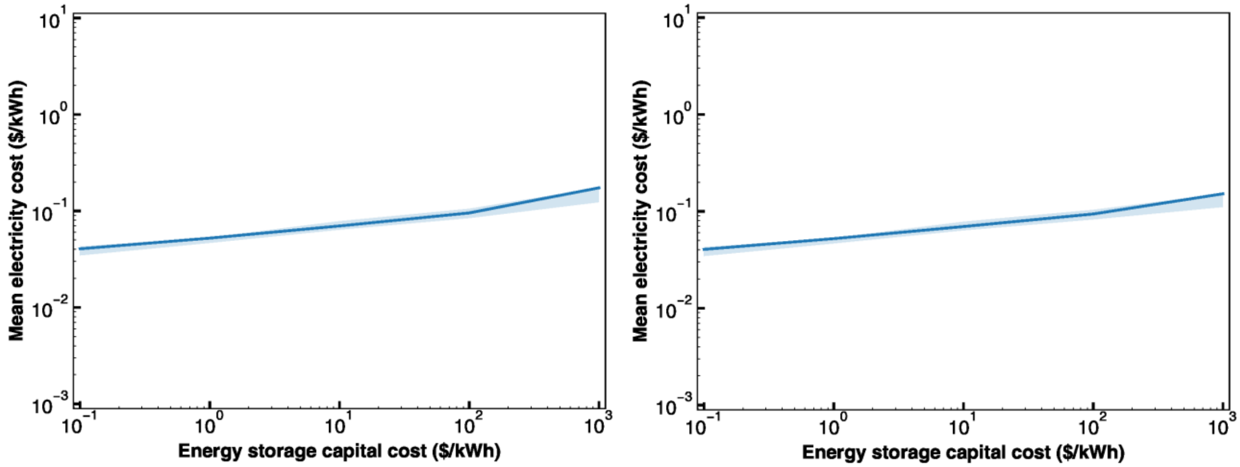

Figure S20. Effect of storage cost on the mean electricity cost of the least-cost VRE/storage systems. The baseline technology scenario (Table S2) is assumed. Bold lines show optimization results for the year 2015, and shaded areas show the range of results from independent annual optimizations (1980–2015). The left panel shows the optimization results for 100% resource adequacy, and the right panel shows those for at least 99.97% resource adequacy. **Related to Figure 1.**

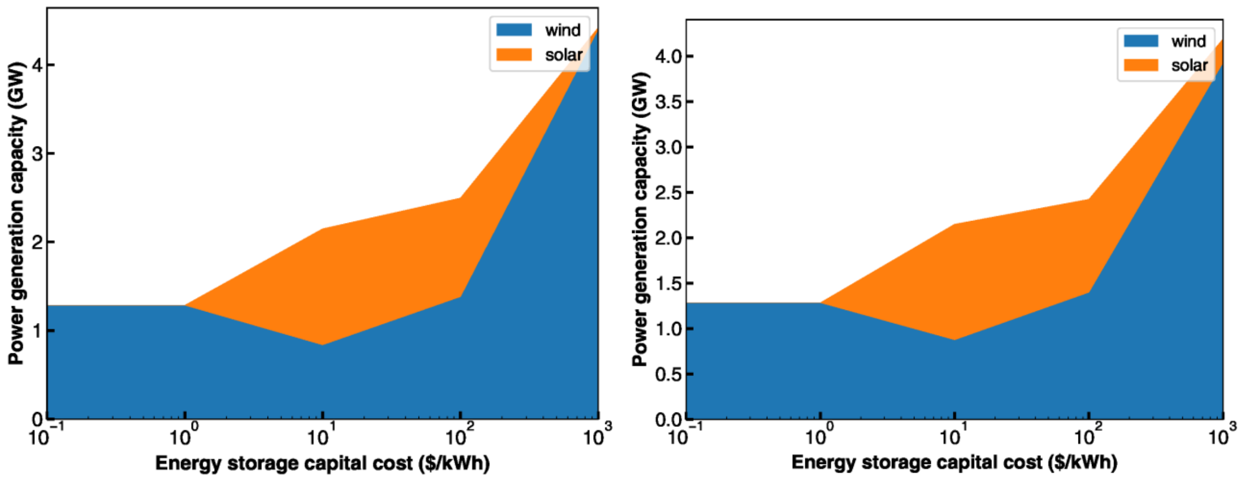

Figure S21. Effect of storage cost on the deployed wind and solar power capacity in the least-cost VRE/storage systems for the year 2015. Results for the deployed energy storage capacity are in Figure S26. The baseline technology scenario (Table S2) is assumed. The left panel shows the optimization results for 100% resource adequacy, and the right panel shows those for at least 99.97% resource adequacy. **Related to Figure 2.**

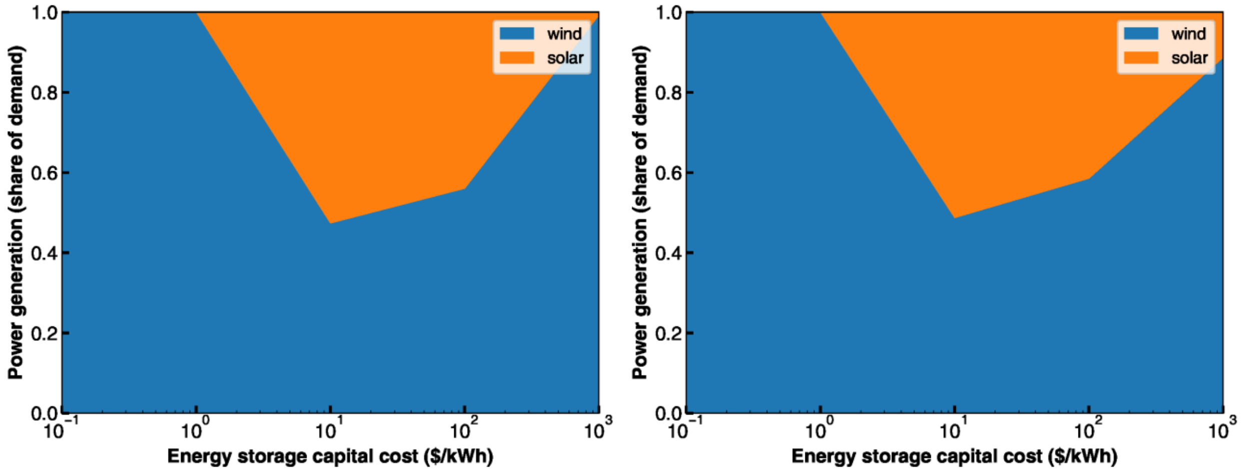

Figure S22. Effect of storage cost on relative shares of net electricity generation in the least-cost VRE/storage systems for the year 2015. Curtailed generation of wind and solar electricity is excluded in this figure but available in Figure S24. The baseline technology scenario (Table S2) is assumed. The left panel shows the optimization results for 100% resource adequacy, and the right panel shows those for at least 99.97% resource adequacy. The total share of wind and solar electricity generation is 99.97% in the right panel. **Related to Figure 2.**

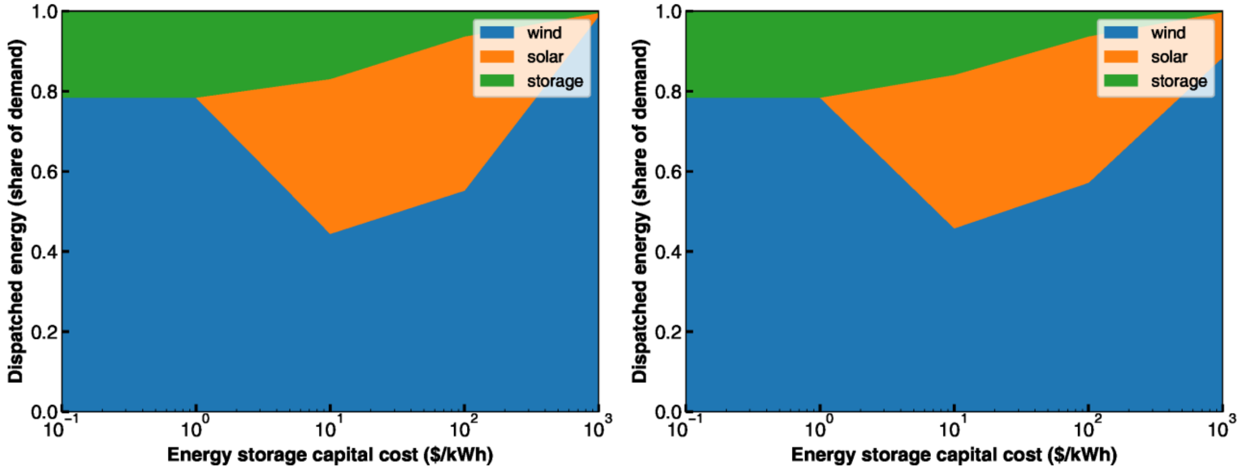

Figure S23. Effect of storage cost on relative shares of dispatched electricity (that directly meets the electricity demand) in the least-cost VRE/storage systems for the year 2015. Curtailed generation of wind and solar electricity are excluded in this figure but available in Figure S24. The baseline technology scenario (Table S2) is assumed. The left panel shows the optimization results for 100% resource adequacy, and the right panel shows those for at least 99.97% resource adequacy. The total share of dispatched energy from wind, solar, and energy storage is 99.97% in the right panel. **Related to Figure 2.**

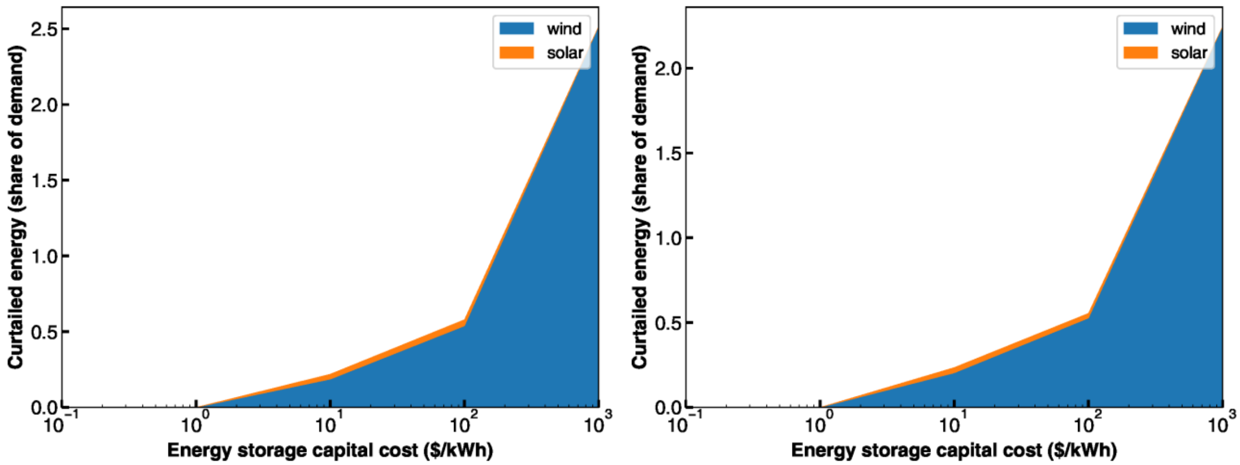

Figure S24. Effect of storage cost on the curtailed generation of wind and solar electricity in the least-cost VRE/storage systems for the year 2015. The baseline technology scenario (Table S2) is assumed. The left panel shows the optimization results for 100% resource adequacy, and the right panel shows those for at least 99.97% resource adequacy. **Related to Figure 2.**

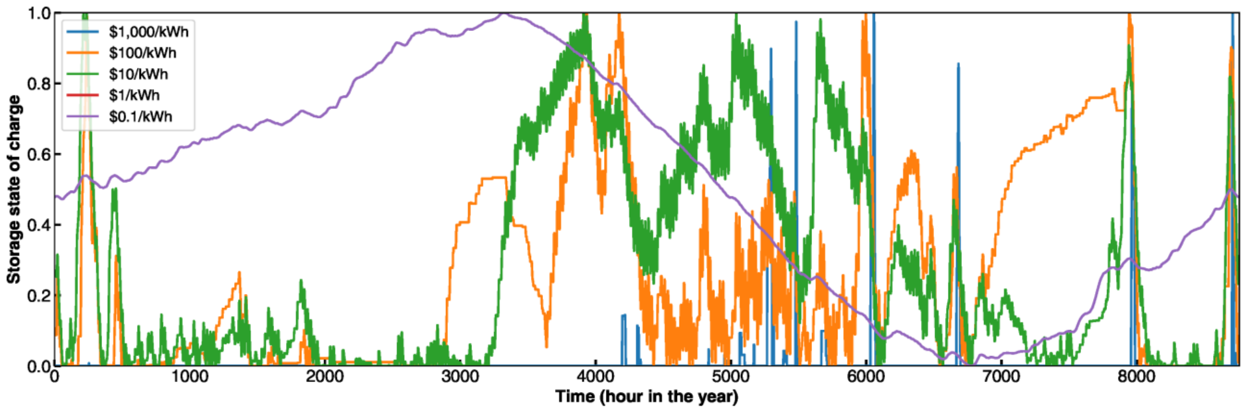

Figure S25. Effect of storage cost on the state-of-charge for energy storage in the least-cost VRE/storage systems for the year 2015. The baseline technology scenario (Table S2) is assumed. This figure is based on the optimization results for at least 99.97% resource adequacy. **Related to Figure 3.**

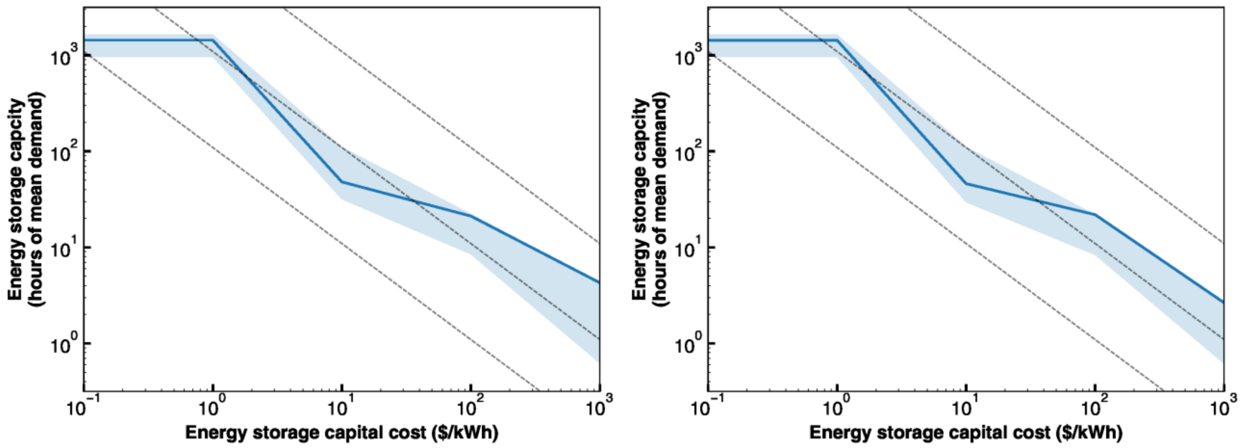

Figure S26. **Effect of storage cost on the deployed storage capacity in the least-cost VRE/storage systems.** Bold lines show optimization results for the year 2015, and shaded areas show the range of results from independent annual optimizations (1980–2015). The left panel shows optimization results for 100% resource adequacy, and the right panel shows those for at least 99.97% resource adequacy. Dashed lines are the same as those shown in Figure 5a. For reference, the mean hourly electricity demand for the US is 449,423 MWh for the year between July 2015 and July 2016. **Related to Figure 5.**

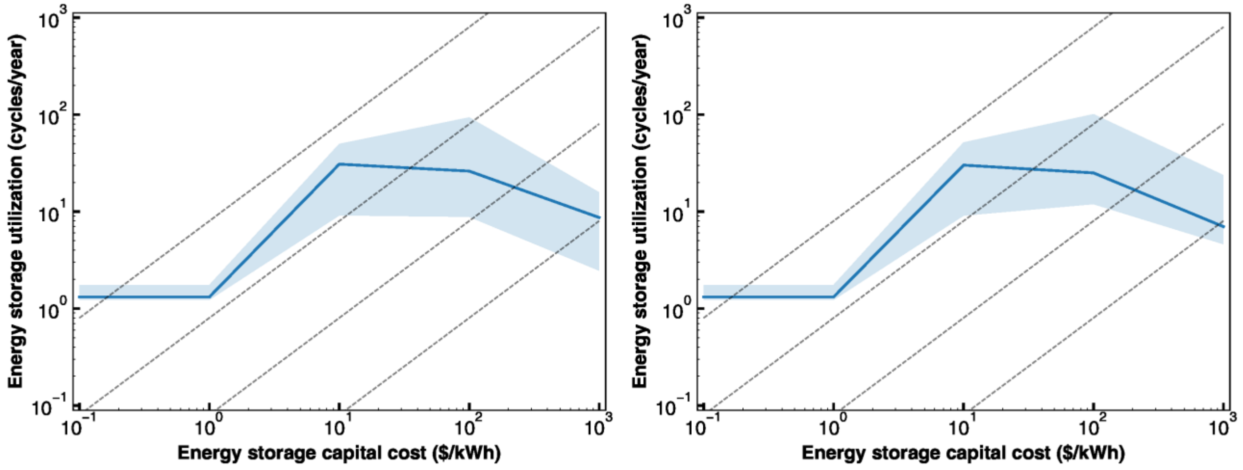

Figure S27. Effect of storage cost on the utilization of storage in the least-cost VRE/storage systems. Bold lines show optimization results for the year 2015, and shaded areas show the range of results from independent annual optimizations (1980–2015). The left panel shows the optimization results for 100% resource adequacy, and the right panel shows those for at least 99.97% resource adequacy. Dashed lines are the same as those shown in Figure 5b. **Related to Figure 5.**

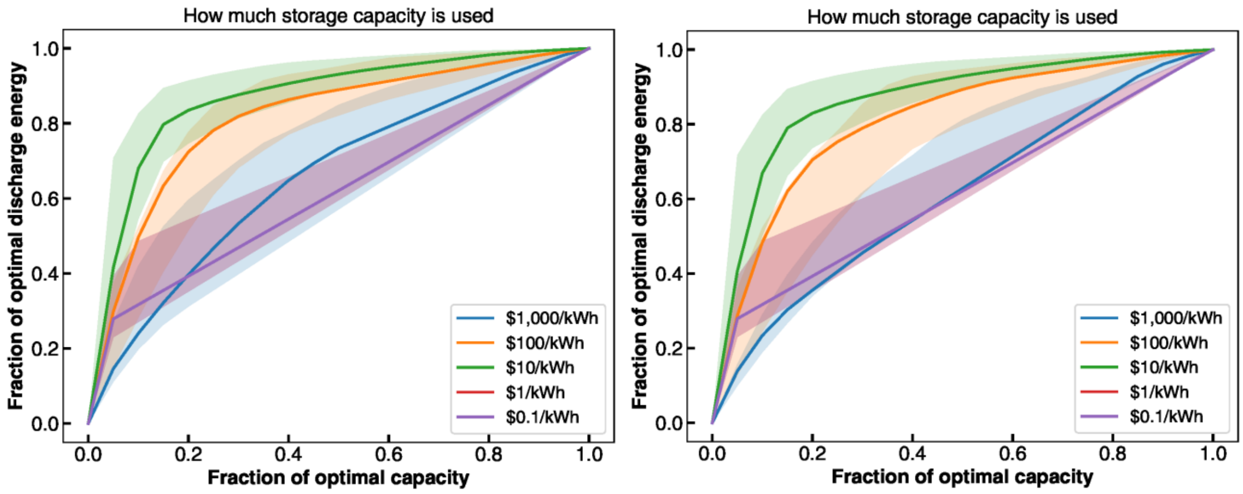

Figure S28. Relationships of discharged energy from storage and storage capacity in the least-cost VRE/storage systems at different storage costs. Bold lines show optimization results for the year 2015, and shaded areas show the range of results from independent annual optimizations (1980–2015). The left panel shows the optimization results for 100% resource adequacy, and the right panel shows those for at least 99.97% resource adequacy. **Related to Figure 6.**

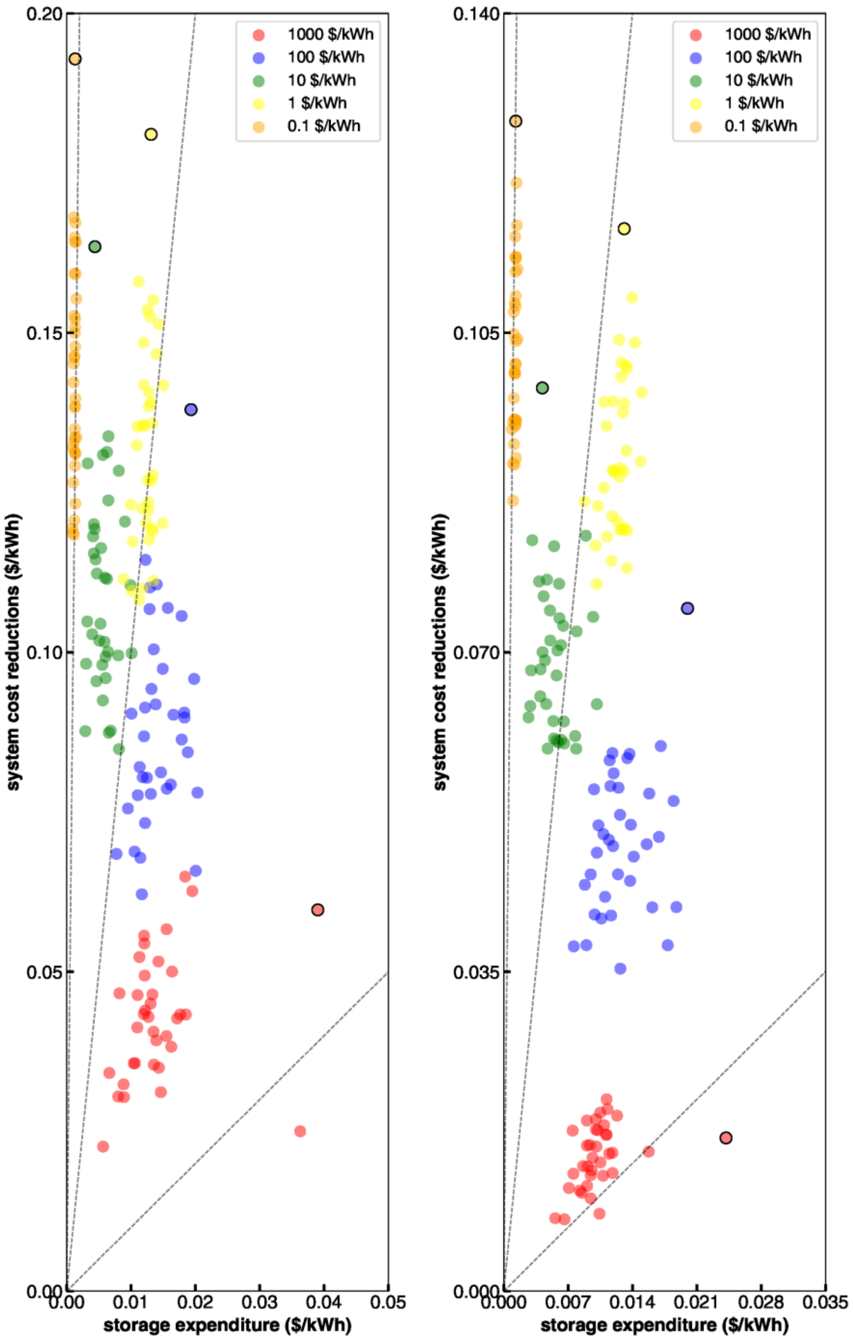

Figure S29. Relationships of system cost reductions and energy storage expenditures for the least-cost VRE/storage systems at different storage costs. Each point represents the results of an independent annual optimization (1980–2015) for an assumed energy storage cost. Points with bold black borders indicate the results for the year 2015. The baseline technology scenario (Table S2) is assumed. The left panel shows the optimization results for 100% resource adequacy, and the right panel shows those for at least 99.97% resource adequacy. **Related to Figure 7.**

Table S1. Technology assumptions for the baseline technology scenario. Related to all figures (Figures 1-7, Figures S1-S29) and tables (Table S1-S5).

Variable	Wind turbine	Solar PV	Energy storage
Capital cost	$1,500/kW	$1,500/kW	$0.1-1,000/kWh
Discount rate	7%	7%	7%
Lifetime (years)	30	30	30
Variable O&M cost ($/kWh)	0	0	0
Fuel cost ($/kWh)	0	0	0
Energy efficiency	-	-	90% (round-trip efficiency)
Capacity factor	38%	22%	-
Charging duration	-	-	1h
Levelized cost of electricity ($/kWh)	0.036	0.062	-

Table S2. Description of technology scenarios considered in this study. Related to all figures (Figures 1-7, Figures S1-S29) and tables (Table S1-S5).

**Scenario name**	**Changes relative to the baseline scenario** (defined in the main text)
Baseline	Wind ($1,500/kW) and solar ($1,500/kW)
Wind only	Only wind ($1,500/kW) is available.
Solar only	Only solar ($1,500/kW) is available.
Wind and cheaper solar	50% reduction in the capital cost of solar power ($750/kW in this scenario)
Cheaper wind and cheaper solar	50% reduction in the capital cost of wind power and solar power (both at $750/kW in this scenario)
Baseline + dispatchable generation (natural gas)	In addition to the technologies in the baseline scenario, we included a dispatchable generation technology, modeled after natural gas combined cycles with carbon capture and storage technology (NGCC-CCS). We assumed a capital cost of $2,200/kW, the fuel cost of $3/MMBtu, and a plant efficiency of 45% for NGCC-CCS (U.S. Energy Information Administration (EIA), 2018a, 2018b).

Table S3. A summary of results for least-cost VRE/storage systems for the baseline technology scenario and the 100% resource adequacy for the year 2015. Related to Figures 2-3 & 5 and Tables S1-S2.

**Energy storage capital cost ($/kWh)**	**Capacity**			**Electricity generation that met the demand**		**Energy storage**		**Curtailment**	
	**Wind (MW)**	**Solar (MW)**	**Storage (MWh)**	**Wind (MWh)**	**Solar (MWh)**	**Charge (MWh)**	**Discharge (MWh)**	**Wind (MWh)**	**Solar (MWh)**
1,000	4.4×10^{6}	1.8×10^{4}	1.9×10^{6}	4.5×10^{5}	3.6×10^{3}	2.1×10^{3}	1.9×10^{3}	1.1×10^{6}	3.1×10^{-13}
100	1.4×10^{6}	1.1×10^{6}	9.5×10^{6}	2.5×10^{5}	2.0×10^{5}	3.2×10^{4}	2.8×10^{4}	2.4×10^{5}	1.9×10^{4}
10	8.4×10^{5}	1.3×10^{6}	2.2×10^{7}	2.2×10^{5}	2.4×10^{5}	8.4×10^{4}	7.6×10^{4}	8.4×10^{4}	1.5×10^{4}
1	1.3×10^{6}	0	6.5×10^{8}	4.6×10^{5}	0	1.1×10^{5}	9.7×10^{4}	-3.6×10^{-11}	0
0.1	1.3×10^{6}	0	6.5×10^{8}	4.6×10^{5}	0	1.1×10^{5}	9.7×10^{4}	-3.6×10^{-11}	0

Table S4. Mean electricity costs and optimal VRE technologies and storage capacities for the least-cost VRE/storage systems at assumed energy storage costs and two resource adequacy criteria (100% and 99.7%) for the year 2015. Related to Figure 1, Figure 5, Figure S19, Figure S21, Figure S26, and Table S3.

Assumptions		Results			
Resource adequacy	Storage cost ($/kWh)	Mean electricity cost ($/kWh)	Wind capacity (MW)	Solar capacity (MW)	Storage capacity (MWh)
100%	1,000	0.17	4.4×10^{6}	1.8×10^{4}	1.9×10^{6}
	100	0.10	1.4×10^{6}	1.1×10^{6}	9.5×10^{6}
	10	0.07	8.4×10^{5}	1.3×10^{6}	2.2×10^{7}
	1	0.05	1.3×10^{6}	0	6.5×10^{8}
	0.1	0.04	1.3×10^{6}	0	6.5×10^{8}
99.97%	1,000	0.15	3.9×10^{6}	2.6×10^{5}	1.2×10^{6}
	100	0.09	1.4×10^{6}	1.0×10^{6}	9.8×10^{6}
	10	0.07	8.8×10^{5}	1.3×10^{6}	2.1×10^{7}
	1	0.05	1.3×10^{6}	0	6.5×10^{8}
	0.1	0.04	1.3×10^{6}	0	6.5×10^{8}

Table S5. The relative difference between optimization results for the 99.7% resource adequacy and those for the 100% resource adequacy for the year 2015. Relative difference is calculated as (result for the 99.97% resource adequacy - result for the 100% resource adequacy) / result for 100% resource adequacy. Related to Figure 1, Figure 5, Figure S19, Figure S21, Figure S26, and Tables S3-S4.

Storage cost	Mean electricity cost	Wind capacity	Solar capacity	Storage capacity
$1,000/kWh	-13%	-11%	1,317%	-38%
$100/kWh	-2%	1%	-8%	3%
$10/kWh	0%	5%	-3%	-4%
$1/kWh	0%	0%	-	0%
$0.1/kWh	0%	0%	-	0%

Supplemental References

Boyd, S., Vandenberghe, L., 2004. Convex Optimization, 1st ed. Cambridge University Press, Cambridge, UK.

Federal Energy Regulatory Commission, 2010. Planning Resource Adequacy Assessment Reliability Standard. San Francisco, CA, United States of America.

National Renewable Energy Laboratory (NREL), 2018. 2018 Annual Technology Baseline. Golden, CO.

Shaner, M.R., Davis, S.J., Lewis, N.S., Caldeira, K., 2018. Geophysical constraints on the reliability of solar and wind power in the United States. Energy Environ. Sci. 11, 914–925.

U.S. Energy Information Administration (EIA), 2017. Hourly Electric Grid Monitor [WWW Document]. URL https://www.eia.gov/beta/electricity/gridmonitor/dashboard/electric_overview/US48/US48

U.S. Energy Information Administration (EIA), 2018a. Annual Energy Outlook 2018. Washington, DC.

U.S. Energy Information Administration (EIA), 2018b. Electric Power Annual 2017. Washington, DC.

Zerrahn, A., Schill, W.-P., 2017. Long-run power storage requirements for high shares of renewables: review and a new model. Renew. Sustain. Energy Rev. 79, 1518–1534.
